# The Plant Pathogen *Phytophthora andina* Emerged via Hybridization of an Unknown *Phytophthora* Species and the Irish Potato Famine Pathogen, *P. infestans*

**DOI:** 10.1371/journal.pone.0024543

**Published:** 2011-09-16

**Authors:** Erica M. Goss, Martha E. Cardenas, Kevin Myers, Gregory A. Forbes, William E. Fry, Silvia Restrepo, Niklaus J. Grünwald

**Affiliations:** 1 Horticultural Crops Research Laboratory, Agricultural Research Service, United States Department of Agriculture, Corvallis, Oregon, United States of America; 2 Laboratorio de Micología y Fitopatología, Universidad de los Andes, Bogotá, Colombia; 3 Department of Plant Pathology and Plant-Microbe Biology, Cornell University, Ithaca, New York, United States of America; 4 International Potato Center, Lima, Peru; Federal University of Rio de Janeiro, Brazil

## Abstract

Emerging plant pathogens have largely been a consequence of the movement of pathogens to new geographic regions. Another documented mechanism for the emergence of plant pathogens is hybridization between individuals of different species or subspecies, which may allow rapid evolution and adaptation to new hosts or environments. Hybrid plant pathogens have traditionally been difficult to detect or confirm, but the increasing ease of cloning and sequencing PCR products now makes the identification of species that consistently have genes or alleles with phylogenetically divergent origins relatively straightforward. We investigated the genetic origin of *Phytophthora andina*, an increasingly common pathogen of Andean crops *Solanum betaceum*, *S. muricatum*, *S. quitoense*, and several wild *Solanum* spp. It has been hypothesized that *P. andina* is a hybrid between the potato late blight pathogen *P. infestans* and another *Phytophthora* species. We tested this hypothesis by cloning four nuclear loci to obtain haplotypes and using these loci to infer the phylogenetic relationships of *P. andina* to *P. infestans* and other related species. Sequencing of cloned PCR products in every case revealed two distinct haplotypes for each locus in *P. andina*, such that each isolate had one allele derived from a *P. infestans* parent and a second divergent allele derived from an unknown species that is closely related but distinct from *P. infestans*, *P. mirabilis*, and *P. ipomoeae*. To the best of our knowledge, the unknown parent has not yet been collected. We also observed sequence polymorphism among *P. andina* isolates at three of the four loci, many of which segregate between previously described *P. andina* clonal lineages. These results provide strong support that *P. andina* emerged via hybridization between *P. infestans* and another unknown *Phytophthora* species also belonging to *Phytophthora* clade 1c.

## Introduction

Emerging plant pathogens threaten natural ecosystems, food security, and commercial interests. Major mechanisms underlying plant pathogen emergence include host range expansion and host jumps [1], [2]. Recently, these events have largely been the result of migration or movement of pathogens or hosts into new geographic regions [3], [4], [5]. Another mechanism is hybridization between species or individuals [6]. Known hybrid plant pathogens include the alder pathogen *Phytophthora alni* [7], the poplar rust *Melampsora*×*columbiana* [8], the crucifer pathogen *Verticillium longisporum* [9], the onion pathogen *Botrytis allii* [10], [11], and *Heterobasidion* forest pathogens [12], [13]. Hybridization and introgression are also hypothesized to be behind the continued epidemic of Dutch elm disease in Europe [14]. Hybridization between a recently introduced exotic pathogen and a resident pathogen may allow rapid evolution and adaptation to new hosts or environments [14], [15], [16], [17], because hybridization introduces genetic variation that has already been “tested by selection” in the resident parental species [18]. The continuing global movement of plant pathogens may be creating opportunities for new and virulent hybrid pathogens to arise [15], [19].

Hybrid plant pathogens have traditionally been difficult to detect or confirm and have generally been investigated for their unusual morphology, pathogenicity, or other phenotypic characters and subsequently identified as hybrids [15], [19]. Modern molecular techniques are currently the gold standard for identifying hybrid pathogens, in particular the sequencing of nuclear loci for which genealogies can be constructed and ancestral and derived states inferred. Based on DNA sequences, hybrids have been identified when sampled individuals consistently have genes or alleles with phylogenetically divergent origins. In diploids or polyploids one may observe that alleles at any one locus are from divergent origins. However, in the case of introgression, when hybrid offspring are not sterile and can backcross to one or the other parental species or strains, the hybridization event may be more difficult to detect if limited DNA sequences are available. Modern molecular methods and especially whole genome sequencing will likely identify additional ‘atypical’ plant pathogens as being hybrids or as having introgressed genes from past hybridization events.

The oomycete pathogen *Phytophthora infestans* is one of the most widely known emerging plant pathogens. It initially emerged in the early 1840s in the United States and Europe and rapidly spread across potato-growing regions, leading to the Irish potato famine. It causes an aggressive disease of potato and tomato, and is still considered a major threat to global food security [20]. In the 1950s, a diverse and sexual population of *P. infestans* was found in the Toluca Valley of central Mexico, on commercial potatoes and then wild relatives of potato, leading to the conventional wisdom that this devastating pathogen evolved in association with the diverse tuber-bearing *Solanum* plant community in the central highlands of Mexico [21], [22]. This scenario is supported by the presence of two closely related species, *P. mirabilis* and *P. ipomoeae*, also found in the Toluca Valley [23], [24]. However, the center of origin and primary center of diversity of potatoes is in the Andean highlands of South America, thus a competing hypothesis is that the Andean highlands are the center of origin of *P. infestans*. This scenario is supported by a genealogical analysis of *P. infestans* using two mitochondrial DNA loci and one nuclear locus that showed old lineages of the pathogen in the Andes and not Mexico [25]. One of the arguments for an Andean origin of *P. infestans* has also been that the closest known relative of *P. infestans*, *P. andina* (formerly known as *P. infestans* sensu lato), is morphologically indistinguishable from *P. infestans* and is found only in the Andean highlands [25], [26]. Furthermore, several apparent lineages of *P. infestans*-like pathogens, all now classified as *P. andina*, has led to the suggestion that the Andes are a hotspot of *Phytophthora* diversification [25].

*Phytophthora andina* was originally discovered when a broader range of blighted *Solanum* species, particularly non-tuber-bearing species, were sampled in Ecuador [26], [27], [28]. These isolates were quickly identified as being genetically distinct from *P. infestans* despite their shared morphology. Specifically, they had new RFLP fingerprints (EC-2 and EC-3) and some EC-2 isolates had a distinct mtDNA haplotype, designated Ic [26], [28]. There are currently three distinct clonal lineages within *P. andina*, defined by RFLP fingerprint (also readily distinguished by AFLP), mitochondrial DNA haplotype, and mating type [26], [29]. Initially these lineages were referred to as *P. infestans* sensu lato, but recently they were all reclassified as *P. andina* Adler & Flier, sp. nov. [29]. Due to the genetic differences among the *P. andina* lineages, this species description is controversial [30]. Host use by *P. infestans* and *P. andina* in Ecuador overlap minimally, with *P. infestans* found infecting *S. tuberosum* (potato), *S. lycopersicum* (tomato), and close relatives (*Solanum* sections Petota, Lycopersicon, and Juglandifolium), and *P. andina* primarily infecting *S. betaceum* (section Pachyphylla), *S. muricatum* (section Basarthrum), *S. quitoense* (section Lasiocarpa), *S. hispidum* (section Torva), and species in the section Anarrhichomenum [29], [31]. Both species have been isolated from *S. muricatum*, *S. quitoense*, and *S. ochranthum* [26], [29]. Genetic variation within *P. andina* may be correlated with host use, suggesting the possibility of host specialization by *P. andina* lineages in the field [26], [29], [31].

*P. infestans* and *P. andina* share identical or nearly identical ITS sequences [29], [32], which is the traditional molecular marker used in species definition in oomycetes and fungi. *P. mirabilis* and *P. ipomoeae* also have identical or nearly identical ITS sequences to *P. infestans* [23]. These four closely related species, plus *P. phaseoli*, make up *Phytophthora* clade 1c [33], [34], [35]. Direct sequencing of nuclear genes in *P. andina* produced identical sequences in all *P. andina* isolates examined [29], [32], but also revealed high levels of heterozygosity with several of these sites differentiating *P. infestans* from *P. mirabilis* sequences [29], [32], [35]. Based on the observed heterozygous sites, it was hypothesized that *P. andina* may be a hybrid between *P. infestans* and *P. mirabilis* [32] or between *P. infestans* and another unspecified parent [29], [35], but the question was not investigated further. Resolution of the ancestry of *P. andina*, particularly whether it is of hybrid origin, is necessary for accurate interpretation of its population structure, evolution, and genetics. Here, we investigate the evolutionary history of *P. andina* and determine whether *P. andina* is in fact a hybrid of *P. infestans* and another species by cloning four nuclear loci to obtain haplotypes to infer the phylogenetic relationships of these alleles in relation to *P. infestans* and related species. Because of the considerable methodological and analytical challenges posed by both the large (∼240 Mb) and highly repetitive (∼74%) *P. infestans* genome [36] and the phasing of haplotypes in short-read, high throughput sequencing approaches, our work relied on traditional PCR cloning of coding sequences.

## Results

**Figure 1:**
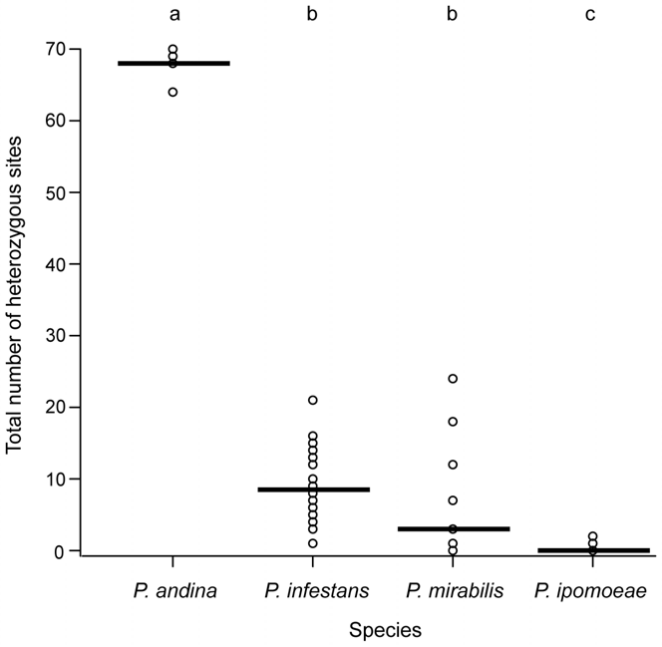
Total number of heterozygous sites across four nuclear loci sequenced in each isolate by species. Lines represent mean values for each species and circles represent values of individual isolates (circles are overlapping). Lowercase letters above graph indicate significance, such that significantly different means (*P*<0.05) by Tukey's HSD are shown by different letters. The number of heterozygous sites observed in *P. andina* isolates was at least two to three times higher than isolates from the other species and *P*-values of comparisons with *P. andina* were less than 0.0001.

Every *P. andina* isolate was heterozygous at each of the four loci sequenced, as evidenced by double peaks in chromatograms from direct sequencing of PCR products. The total number of heterozygous sites summed across the four sequenced loci was significantly higher in *P. andina* compared to *P. infestans*, *P. ipomoeae*, and *P. mirabilis* (Figure 1; *P*<0.0001 for each comparison with *P. andina* by Tukey HSD). On average, *P. andina* isolates had greater than seven times more heterozygous sites than the other three species (Figure 1). Heterozygosity for indels was also observed in both regions of *ypt1*, *btub* and PITG11126, such that chromatograms showed overlapping PCR products of different lengths. Heterozygosity was observed in *P. infestans*, *P. mirabilis*, and in one isolate of *P. ipomoeae*, but with many fewer heterozygous sites per locus. When maximum likelihood gene trees were constructed using genotypes, *P. andina* could not be distinguished from *P. infestans* (Figure S1).

**Figure 2:**
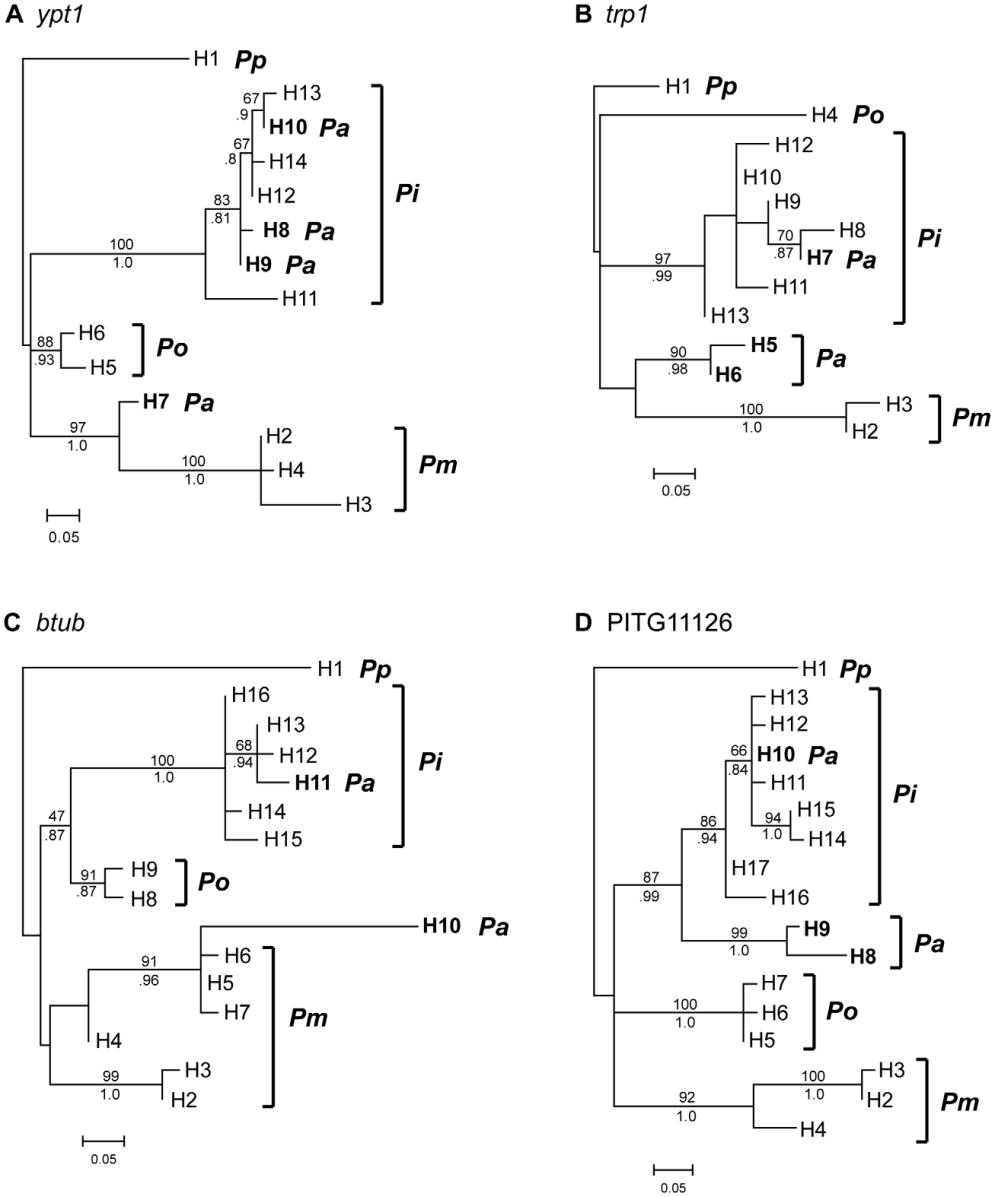
Maximum likelihood trees of haplotypes for each locus sequenced. Loci are **A**. *ypt1*, **B**. *trp1*, **C**. *btub*, and **D**. PITG11126, sequenced in *P. andina* (***Pa***) and four other closely related species (***Pi***: *P. infestans*; ***Pm***: *P. mirabilis*; ***Po***: *P. ipomoeae*; and ***Pp***: *P. phaseoli*). The haplotype designation is shown for each branch tip, corresponding to Tables 1, 2, and S1, S2, S3, S4, S5, S6, S7, S8. *P. andina* haplotypes are bolded. Trees have been rooted with *P. phaseoli*. Bootstrap support values obtained by maximum likelihood are shown above branches and Bayesian posterior probabilities are shown below branches. Values are not shown for branches that had less then 80% support/probability by both methods.

Sequencing of cloned PCR products in every case revealed two distinct haplotypes for *P. andina* isolates (Table 1). For *btub*, *trp1*, and PITG11126, one haplotype was identical to the most common *P. infestans* haplotype, found in isolates from the Andes, the United States, Mexico, and the United Kingdom (Table S1). For *ypt1*, *P. andina* isolates had one of two *P. infestans* haplotypes (H9 or H10) differing by 2 bp, with the exception of EC_3678, which had a *P. infestans*-like haplotype (H8) that differed from H9 at one nucleotide site (Table S2A). The second haplotype in each isolate was more or less distantly related to *P. infestans* depending on the locus (Figure 2). There were two versions of the non-*P. infestans* haplotypes for *trp1* and PITG11126, which differed by 1 and 5 bp, respectively (Table S2). Much of the observed variation within *P. andina* segregates between the three *P. andina* lineages (Table 2).

**Table 1:** *P. andina* isolates and haplotypes of each locus sequenced.

			Haplotypes^a^				
Isolate	Origin	Host	mtDNA^b^	*ypt1*	*trp1*	*btub*	PITG11126
EC 3163	Ecuador	Anarrichomenum group	Ic	H7/H9	H5/H7	H10/H11	H8/H10
EC 3189	Ecuador	Anarrichomenum group	Ic	H7/H10	H5/H7	H10/H11	H8/H10
EC 3399	Ecuador	Anarrichomenum group	Ia	H7/H10	H5/H7	H10/H11	H8/H10
EC 3510	Ecuador	*S. betaceum*	Ia	H7/H9	H6/H7	H10/H11	H9/H10
EC 3540	Ecuador	Anarrichomenum group	Ic	H7/H9	H5/H7	H10/H11	H8/H10
EC 3561	Ecuador	*S. quitoense*	Ia	H7/H10	H5/H7	H10/H11	H8/H10
EC 3563	Ecuador	*S. quitoense*	Ia	H7/H10	H5/H7	H10/H11	H8/H10
EC 3655	Ecuador	*S. hispidum*	Ic	H7/H9	H5/H7	H10/H11	H8/H10
EC 3678	Ecuador	Anarrichomenum group	Ic	H7/H8	H5/H7	H10/H11	H8/H10
EC 3780	Ecuador	*S. hispidum*	Ic	H7/H9	H5/H7	H10/H11	H8/H10
EC 3818	Ecuador	Anarrichomenum group	Ia	H7/H10	H5/H7	H10/H11	H8/H10
EC 3821	Ecuador	Anarrichomenum group	Ia	H7/H10	H5/H7	H10/H11	H8/H10
EC 3836	Ecuador	*S. betaceum*	Ia	H7/H9	H6/H7	H10/H11	H9/H10
EC 3860	Ecuador	Torva group	Ic	H7/H9	H5/H7	H10/H11	H8/H10
EC 3864	Ecuador	Torva group	Ic	H7/H9	H5/H7	H10/H11	H8/H10
POX 102	Peru	*S. betaceum*	Ic	H7/H9	H5/H7	H10/H11	H8/H10
POX 103	Peru	*S. betaceum*	Ic	H7/H9	H5/H7	H10/H11	H8/H10

a Haplotype designations for other species are given in Table S1.

b Haplotype designations for *P. andina* as described in references [28], [58].

**Table 2:** Summary of sequence variation among *P. andina* lineages.

			*P. andina* lineage^a^		
Locus	Variable allele	Segregating sites	EC2 Ia	EC2 Ic	EC3 Ia
*ypt1*	*P. infestans*	2^b^	H10	H8^c^, H9, H10^c^	H9
*trp1*	Non-*P. infestans*	1	H5	H5	H6
PITG11126	Non-*P. infestans*	5	H8	H8	H9

a *P. andina* lineage is given as the RG57 genotype followed by the mtDNA haplotype.

b Two differences between H9 and H10. H8 had one difference from H9 and three from H10.

c One EC2 Ic isolate had *P. infestans* haplotype H10 and one had a *P. infestans*-like haplotype H8.

The non-*P. infestans P. andina* haplotypes (hereafter *Pa*-unknown) for *ypt1*, *btub*, and *trp1*, were related to *P. mirabilis*, but clearly distinct. Different possible phylogenetic relationships among *Pa*-unknown, *P. infestans*, *P. mirabilis*, and *P. ipomoeae* were statistically tested using the approximately unbiased (AU) test and Shimodaira–Hasegawa (SH) tests (Table S9). All trees were essentially star phylogenies for *trp1*, thus the AU test failed and no trees were rejected by the SH test. For *ypt1*, trees that did not contain a derived {*Pa*-unknown, *P. mirabilis*} clade had low *P* values by the AU and SH tests (0.05<*P*<0.1), but no trees were rejected at the *P* = 0.05 level. For *btub*, trees with the complex {*Pa*-unknown, *P. mirabilis*} clade had high *P* values. But one tree with monophyletic *P. mirabilis* as sister species to *Pa*-unknown was also not rejected, as well as two trees in which *P. infestans* and *P. ipomoeae* formed a derived clade (*P*>0.1 by the AU test, 0.05<*P*<0.1 by the SH test). Species relationships were qualitatively different for the PITG11126 locus. The *Pa*-unknown haplotype was more closely related to *P. infestans* than *P. mirabilis* (Fig. 2D). Unlike the other loci, sites in PITG11126 that differed between *P. infestans* and *P. phaseoli*, *P. mirabilis*, and *P. ipomoeae* were in the *P. infestans* state in *P. andina* (Table S2D). The AU test rejected all trees that did not include a derived {*Pa*-unknown, *P. infestans*} clade or a derived {*Pa*-unknown, *P. ipomoeae*} clade.

## Discussion

We tested the hypothesis that *Phytophthora andina* is a hybrid pathogen and found that it is a hybrid between *P. infestans* and an unknown species that is closely related but distinct from *P. mirabilis* and *P. ipomoeae*. Cloning and sequencing of four nuclear loci clearly shows that *P. andina* isolates have one allele derived from a *P. infestans* parent and a second divergent allele from the unknown species. The *P. infestans* haplotypes found in *P. andina* appear to be common worldwide, found in North and South America, Europe, and Asia. Because *P. andina* has a different host range than *P. infestans* and the three *P. andina* lineages may have different host ranges themselves [26], [29], [31], it is probable that hybridization led to host range expansion or shifts.

Host shifts are likely to require several rapid genetic changes, but *P. andina* may have been in a unique position to undergo the necessary changes and rapidly adapt to new hosts. First, hybridization may facilitate adaptation to a new environment by rapidly introducing genetic variation, and not random variation but rather a complement of alleles that have been subjected to selection in the parental species [18]. Second, *P. infestans* has a genome structure which may have contributed to its ability to rapidly evolve virulence to resistant potato varieties in the near absence of sexual reproduction [36]. In particular, *P. infestans* has a very large genome with expanded repeat-rich gene-sparse regions where pathogenicity effectors, genes involved in virulence and host range, are primarily located. Comparisons to the genome sequences of two distantly related *Phytophthora* species show considerable expansion of effector genes in *P. infestans* and suggest that the repeat-rich gene-sparse regions are highly dynamic, exhibiting gene duplications and gene loss by tandem duplication, non-allelic homologous recombination, and pseudogenization. *P. ipomoeae*, *P. mirabilis*, and *P. phaseoli* have similar genome structures to *P. infestans* and comparisons among these species show greater gene copy number variation and presence/absence polymorphisms in the repeat-rich regions compared to the gene-dense repeat-poor regions where core orthologs are found [37]. The repeat-rich regions are also enriched in genes induced during infection. *P. infestans* is also known to exhibit aneuploidy, particularly in the clonal lineages that dominate much of its current geographic distribution [38], [39], [40]. Thus, *P. andina* had a potential mechanism for rapid change in its genic and allelic composition following hybridization. Different evolutionary paths taken by hybrid progeny could also explain the genetic variation observed within *P. andina*.

We cloned parental haplotypes at different frequencies from *P. andina* isolates for several loci, and while it is possible that *P. andina* is tetraploid or aneuploid and that the haplotypes are actually present in *P. andina* at different frequencies, it is more likely that there is a bias in the efficiency of the primers between haplotypes. The primers were designed from *P. infestans* and may contain mismatches with the sequences of the other *Phytophthora* clade 1c species. Cloned recombinant haplotypes are likely to be chimeras from PCR error, as PCR conditions were not optimized to reduce these sorts of errors [9], [41]. Illumina sequence reads from *P. andina* graciously shared with us [S. Kamoun personal communication, [37]] were examined, but these data could not be analyzed because depth of coverage was not sufficient to call heterozygous sites with high confidence [42] and the short reads were problematic for determining haplotype phase. More extensive deep sequencing may elucidate the genome composition of *P. andina*, particularly using sequencing technologies that generate longer read lengths. Genome-wide analysis would also allow for examination of alternate hypotheses for the observed pattern of phylogenetically distinct alleles at each locus, including mechanisms such as gene duplication or horizontal gene transfer.

The hybrid *P. alni*, a lethal pathogen of alder, is another example of hybridization between closely related *Phytophthora* species resulting in a host range expansion or shift [7], [43], [44]. Additional examples include hybrid *Phytophthora* pathogens causing disease on loquat trees in Peru and Taiwan [45], [46] and in ornamental nurseries where exotic pathogens are brought together under artificial conditions [47], [48], [49]. Host range expansions by *Phytophthora* hybrids have been documented for both these naturally occurring hybrids and for hybrids created in the laboratory [6]. *P. infestans* and *P. mirabilis* are outcrossers, occur in sympatry on different hosts in the Toluca Valley of central Mexico, and are thought to have evolved by sympatric speciation via host shifts [21], thus the potential for interspecific mating between these species has been investigated. Population genetic analysis suggested some gene flow between *P. infestans* and *P. mirabilis* populations [21], [50]. However, initial crosses between *P. infestans* and *P. mirabilis* produced hybrid offspring that were largely unable to infect either host group and had poor viability [51]. Nevertheless, a recent cross between a *P. infestans* isolate (virulent on potato and tomato) and a *P. mirabilis* isolate (virulent on *Mirabilis jalapa*), both from central Mexico, produced F1 and F2 progeny that were pathogenic on tomato and one F2 isolate had an expanded host range, able to infect all parental hosts [52]. Interestingly, the ability to infect tomato segregated as a dominant single locus trait in this cross. Sexual crosses have also been attempted between *P. infestans* and *P. andina* [53]. A limited number of viable progeny were obtained, but further crosses with these progeny were not successful. Here we examined only four nuclear loci, yet we observed both parental haplotypes at each locus for each isolate, which suggests that these *P. andina* lineages were not the result of backcrosses.

Reproductive barriers between closely related species are usually stronger when the species occur in sympatry than when the species have evolved in allopatry [54]. There is not yet strong evidence for this pattern specifically for fungal or oomycete pathogens, in part because the native distributions of many of these pathogens are not well known. Essentially, it is not clear where pathogens evolved and therefore whether sister species evolved in sympatry or allopatry. On the other hand, host shift speciation may also occur without intrinsic reproductive barriers when pathogens must sexually reproduce on their host [1]. It has nevertheless been hypothesized that *Phytophthora* hybrids are offspring of two exotic species or of an exotic and resident species [6], [48]. If this pattern does hold true for *Phytophthora*, it would suggest that at least one of the *P. andina* parent species is introduced and did not co-evolve with the Andean *Solanum* host community [52].

Synthetic hybridization experiments have been used to recreate hybrids, to a certain extent, observed in the wild in order to validate the hybrid origin of species (e.g. [18], [55], [56]). For *P. andina*, one of the hybrid parents remains unknown and so these experiments remain to be conducted, pending the collection and identification of the unknown parent. However, locating this species could be challenging. Disease epidemics caused by *P. mirabilis* and *P. ipomoeae* are infrequent and incidence of infection is low (N. J. Grünwald, personal observation). This would probably also be true of other relatives of *P. infestans* that infect wild and patchy host populations. The unknown species suggests that there is undiscovered diversity in *Phytophthora* clade 1c that may be found in the Andes, although the evolutionary origin of this species in relationship to *P. infestans* and its Mexican sister species remains unclear.

*Phytophthora* diseases are currently being managed with fungicides or, preferably, resistant plant varieties when available. Global movement and interspecific hybridization of plant pathogens multiply the considerable challenges already faced by crop breeding programs and growers trying to manage disease. The global movement of plant pathogens may increase the risk of formation of novel hybrid *Phytophthora* pathogens if hybridization is more likely between previously allopatric species brought together by migration events. Understanding the ecological and genetic processes that result in hybrid pathogens with novel host ranges or virulence, as appears to be the case for *P. andina*, should suggest conditions under which special vigilance and increased monitoring for emerging pathogens is warranted.

## Materials and Methods

### Isolates

Isolates or genomic DNA of clade 1c species were kindly provided by several researchers (Table S1). *P. andina* was distinguished from *P. infestans* based on the host from which isolates were collected, an apparently complementary mating system, AFLP markers, and sequence differences at in *Ras* intron 1 gene [29]. Isolates were received as genomic DNA or maintained on Rye A agar [57]. Total genomic DNA was extracted from mycelium grown in pea broth (*P. infestans* and *P. andina*) or clarified V8 (other species) using the FastDNA SPIN kit (MP Biomedicals LLC, Solon, OH).

### mtDNA RFLP

Mitochondrial DNA haplotype sensu Griffith and Shaw [58] was determined for each *P. andina* isolate by amplifying and digesting the P2 and P4 regions as described by Griffith and Shaw.

### Nuclear gene sequencing

The following genes known to contain variation within and among *Phytophthora* species were chosen for sequencing: the Ras gene *ypt1* [25], [59]; *trp1*, *btub*, [33], [35], [60], and an additional gene that also exhibited variation within and among 1c species in preliminary sequencing (PITG11126, [61]). Primers for *ypt1* were from Gomez-Alpizar et al. [25]. These amplify a fragment of the 5′ untranslated region of the gene (intron1, IR) and a larger downstream fragment including both exons and introns (RAS). These two fragments were concatenated for analysis. Primers for the other genes were designed from the *P. infestans* genome sequence [36] (Table 3). All isolates were directly sequenced from the PCR product. For each locus, two to six *P. andina* isolates were selected for cloning of the PCR product to obtain haplotypes (Table 3). For *ypt1*, 6 isolates were additionally cloned across the entire region to obtain haplotypes across both amplified fragments. Several *P. infestans* and *P. mirabilis* isolates with heterozygous sites were also cloned to obtain haplotypes. When a chromatogram indicated that the isolate was heterozygous for an indel at a sequenced locus, the preliminary sequence was determined using the sequence obtained from each primer up to the indel (i.e. sequence was inferred from one strand). Then, isolates representing each inferred genotype were cloned to obtain haplotypes and confirm the genotype inferred from direct sequencing. Specific cloning and sequencing methods and protocols differed among the laboratories (Fry, Grünwald, Restrepo) where they were performed and are available upon request (see also [60], [62]).

**Table 3:** Loci sequenced and *P. andina* isolates cloned.

Locus	Length^a^	*Pa* isolates cloned	Primers	T_a_ b
*ypt1* (IR)	227	EC 3399, EC 3561, EC 3563, EC 3818, EC 3821, EC 3189	IRF – TTGCAGCACAACCCAAGACG; IRR – TGCACGTACTATTCGGGGTTC	61C
*ypt1* (RAS)	544	EC 3163, EC 3399, EC 3563, EC 3821	RASF – CGTGTCTGCTTCTCCGTTTCG; RASR – CCAGGCTTTCGGCAAATTCC	61C
*ypt1* (IRRAS)	987	EC 3163, EC 3510, EC 3563, EC 3655, EC 3818, POX 102	IRF – TTGCAGCACAACCCAAGACG; RASR – CCAGGCTTTCGGCAAATTCC	61C
*trp1*	814	EC 3818, POX 102	F3 – GGGTAACATCCTGGAGGAGA; R3 - TCGTACTTGACCACGTCTGC	63C
beta-tubulin	1592	EC 3836, POX 102	F1 – GTCCGAATTCTCCTCAGAGC; F2 – CGCTATCGGTACACCAGCTT; F3b – ACCATAACGAAGGGAAAGG; R1 – GATGCCAAGCCACTAACCTC; R2 – CCTCATTGTCCAGGCACAT	58C
PITG11126	788	EC 3163, EC 3510, POX 102	F1 – GGGGACTTCGCTGTTTGTTA; R1 – ATGTTCATGTACGGCTGACG	59C

a Length of the multiple sequence alignment across all sequenced isolates.

b Optimal annealing temperature of primers for PCR based on experience in the Grünwald lab.

The number of heterozygous sites was summed across the four sequenced loci for each isolate for which sequence was obtained for all loci. This total per isolate was used to examine differences in the number of heterozygous sites among species using analysis of variance, implemented in R 2.11.1 for Mac OS. Post-hoc multiple comparisons were conducted using Tukey's HSD.

### Haplotype inference

More than two haplotypes were often obtained from cloning *P. andina* isolates (Tables S3, S4, S5, S6, S7, S8). Haplotypes that were common across isolates were inferred to be the non-recombinant (parental) haplotypes. Other haplotypes cloned from *P. andina* were recombinants of the two parental haplotypes and were not included in the analyzed data sets. For some loci and *P. andina* isolates, the inferred parental haplotypes were cloned at unequal frequencies (Tables S3 and S6, S7, S8).

Haplotypes for each *P. infestans* isolate were inferred from genotypes using PHASE v2.1 [63], [64]. Selected isolates were cloned to confirm inferred haplotypes. When the cloned sequences did not match the inferred haplotypes because the genotype was a combination of three alleles (*btub* in two Colombian isolates), all three alleles were included in the data set. When the inferred haplotypes were recovered by cloning, but additional recombinant haplotypes were also cloned, the recombinant haplotypes were not included in the analyzed sequences. All haplotypes included in the analysis were submitted to Genbank (Accession numbers JF919525–JF919609). Recombinant haplotypes are provided as supporting data.

### Phylogenetic methods

Sequences were aligned using ClustalW [65]. Sequence alignments were collapsed to unique haplotypes, removing invariable sites and indels using Map in SNAP WORKBENCH [66], [67]. Jmodeltest [68] was used to estimate a nucleotide substitution model using maximum-likelihood trees estimated for each model and model selection by AIC.

Maximum likelihood (ML) gene trees were inferred using PhyML [69], implemented in Geneious 5.0.2 (Biomatters Ltd.), using the substitution model selected by jmodeltest (HKY for *trp1*, GTR for *ypt1*, *btub*, and PITG11126). The transition/transversion ratio, proportion of invariable sites, and gamma distribution parameter were estimated from the data in PhyML using 6 rate categories. Data sets were bootstrapped using 500 samples.

Gene trees were also inferred using MrBayes [70], implemented in Geneious 5.0.2. The same nucleotide substitution model was used as for PhyML. MCMC used 4 heated chains of 1.1×10^6^ steps sampled every 200 steps. Posterior trees were summarized excluding the initial 500 trees as burn-in. The default priors were used.

The approximately unbiased (AU) test of Shimodaira [71] and Shimodaira–Hasegawa (SH) test [72] was used to test among tree topologies using the program CONSEL [73]. We tested 15 topologies for each locus, in which the phylogenetic relationships among *P. infestans*, *P. ipomoeae*, *P. mirabilis*, and the non-*P. infestans* parent of *P. andina* (*Pa*-unknown) were varied. All trees were rooted with *P. phaseoli*. Site likelihoods were estimated in PhyML as described above with the exception that the topology was set to the input tree and not optimized. Three additional trees were tested for *btub*, for which ML and Bayesian trees showed *P. mirabilis* forming two clades with *Pa*-unknown embedded in one of these clades. Monophyly of *P. mirabilis* was forced in 15 trees while three additional trees tested the relative relationship of the inferred complex {*P. mirabilis*, *Pa*-unknown} clade to *P. infestans* and *P. ipomoeae*.

## Supporting Information

Figure S1 **Maximum likelihood trees of genotypes for each locus sequenced in** ***P. andina*** **and four other closely related species.** Loci are **A**. *ypt1*, **B**. *trp1*, **C**. *btub*, and **D**. PITG11126. Genotypes are shown as combinations of haplotypes. Bootstrap support values obtained by maximum likelihood are shown above branches.
(TIF)

Table S1 **Isolates and haplotype designations for each locus sequenced.**
(DOCX)

Table S2 **Variable sites at each sequenced locus: A.** ***ypt1*** **, B.** ***trp1*** **, C.** ***btub*** **, and D. PITG11126.** For each locus, the consensus sequence across clade 1c species is shown and identity to this sequence indicated with a dot. Haplotype numbers for each locus correspond with those in Tables 1, 2, and S1, and Figure 2. Site numbers indicate position in multispecies alignment. Indels are not included; see Tables S3, S4, S5, S6, S7, S8 for indels that are heterozygous in *P. andina*. Sites with shared nucleotides between the non-*P. infestans* haplotype in *P. andina* and *P. mirabilis*, *P. ipomoeae*, or *P. infestans* are shown in bold.
(DOCX)

Table S3 ***P. andina ypt1*** **haplotypes obtained from cloning for the full region (IR through RAS).** Italicized sites are between sequenced regions and were not included in the analysis.
(DOCX)

Table S4 ***P. andina*** **haplotypes obtained from cloning just the IR region, including those shown in Table S3.**
(DOCX)

Table S5 ***P. andina*** **haplotypes obtained from cloning just the RAS region, including those shown in Table S3.**
(DOCX)

Table S6 ***P. andina trp1*** **haplotypes obtained from cloning.**
(DOCX)

Table S7 ***P. andina btub*** **haplotypes obtained from cloning.**
(DOCX)

Table S8 ***P. andina*** **PITG11126 haplotypes obtained from cloning.**
(DOCX)

Table S9 **Results of AU and SH tests of tree topologies for** ***ypt1*** **,** ***btub*** **, and PITG11126.**
(DOCX)

## References

[1] Giraud T, Gladieux P, Gavrilets S (2010). Linking the emergence of fungal plant diseases with ecological speciation.. Trends in Ecology & Evolution.

[2] Stukenbrock EH, McDonald BA (2008). The origins of plant pathogens in agro-ecosystems.. Annual Review of Phytopathology.

[3] Anderson PK, Cunningham AA, Patel NG, Morales FJ, Epstein PR (2004). Emerging infectious diseases of plants: pathogen pollution, climate change and agrotechnology drivers.. Trends in Ecology & Evolution.

[4] Brasier CM (2008). The biosecurity threat to the UK and global environment from international trade in plants.. Plant Pathology.

[5] Brown JKM, Hovmoller MS (2002). Aerial dispersal of pathogens on the global and continental scales and its impact on plant disease.. Science.

[6] Ersek T, Nagy ZA (2008). Species hybrids in the genus *Phytophthora* with emphasis on the alder pathogen *Phytophthora alni*: a review.. European Journal of Plant Pathology.

[7] Brasier CM, Cooke DEL, Duncan JM (1999). Origin of a new *Phytophthora* pathogen through interspecific hybridization.. Proceedings of the National Academy of Sciences, USA.

[8] Newcombe G, Stirling B, McDonald S, Bradshaw HD (2000). *Melampsora*×*columbiana*, a natural hybrid of *M. medusae* and *M. occidentalis*.. Mycological Research.

[9] Inderbitzin P, Davis RM, Bostock RM, Subbarao KV (2011). The ascomycete *Verticillium longisporum* is a hybrid and a plant pathogen with an expanded host range.. PLoS ONE.

[10] Nielsen K, Yohalem DS (2001). Origin of a polypoloid *Botrytis* pathogen through interspecific hybridization between *Botrytis aclada* and *B. byssoidea*.. Mycologia.

[11] Staats M, van Baarlen P, van Kan JAL (2005). Molecular phylogeny of the plant pathogenic genus *Botrytis* and the evolution of host specificity.. Mol Biol Evol.

[12] Garbelotto M, Ratcliff A, Bruns TD, Cobb FW, Otrosina WJ (1996). Use of taxon-specific competitive-priming PCR to study host specificity, hybridization, and intergroup gene flow in intersterility groups of *Heterobasidion annosum*.. Phytopathology.

[13] Gonthier P, Nicolotti G, Linzer R, Guglielmo F, Garbelotto M (2007). Invasion of European pine stands by a North American forest pathogen and its hybridization with a native interfertile taxon.. Molecular Ecology.

[14] Brasier CM (2001). Rapid evolution of introduced plant pathogens via interspecific hybridization.. BioScience.

[15] Brasier CM (2000). The rise of the hybrid fungi.. Nature.

[16] Giraud T, Refregier G, Le Gac M, de Vienne DM, Hood ME (2008). Speciation in fungi.. Fungal Genetics and Biology.

[17] Schardl CL, Craven KD (2003). Interspecific hybridization in plant-associated fungi and oomycetes: a review.. Molecular Ecology.

[18] Reiseberg LH, Raymond O, Rosenthal DM, Lai Z, Livingstone K (2003). Major ecological transitions in wild sunflowers facilitated by hybridization.. Science.

[19] Yan S, Liu H, Mohr TJ, Jenrette J, Chiodini R (2008). Role of recombination in the evolution of the model plant pathogen *Pseudomonas syringae* pv. tomato DC3000, a very atypical tomato strain.. Applied and Environmental Microbiology.

[20] Pennisi E (2010). Armed and Dangerous.. Science.

[21] Grünwald NJ, Flier WG (2005). The biology of *Phytophthora infestans* at its center of origin.. Annual Review of Phytopathology.

[22] Grünwald NJ, Flier WG, Sturbaum AK, Garay-Serrano E, van den Bosch TBM (2001). Population Structure of *Phytophthora infestans* in the Toluca Valley Region of Central Mexico.. Phytopathology.

[23] Flier WG, Grünwald NJ, Kroon LPNM, van den Bosch TBM, Garay-Serrano E (2002). *Phytophthora ipomoeae* sp. nov., a new homothallic species causing leaf blight on *Ipomoea longipedunculata* in the Toluca Valley of central Mexico.. Mycological Research.

[24] Galindo J, Hohl HR (1985). *Phytophthora mirabilis*, a new species of *Phytophthora*.. Sydowia.

[25] Gomez-Alpizar L, Carbone I, Ristaino JB (2007). An Andean origin of *Phytophthora infestans* inferred from mitochondrial and nuclear gene genealogies.. Proceedings of the National Academy of Sciences, USA.

[26] Adler NE, Erselius LJ, Chacon MG, Flier WG, Ordonez ME (2004). Genetic diversity of *Phytophthora infestans* sensu lato in Ecuador provides new insight into the origin of this important plant pathogen.. Phytopathology.

[27] Oyarzun PJ, Ordonez ME, Forbes GA (1997). First report of *Phytophthora infestans* A2 mating type in Ecuador.. Plant Disease.

[28] Ordonez ME, Hohl HR, Velasco JA, Ramon MP, Oyarzun PJ (2000). A novel population of *Phytophthora*, similar to *P. infestans*, attacks wild *Solanum* species in Ecuador.. Phytopathology.

[29] Oliva RF, Kroon LPNM, Chacon G, Flier WG, Ristaino JB (2010). *Phytophthora andina* sp. nov., a newly identified heterothallic pathogen of solanaceous hosts in the Andean highlands.. Plant Pathology.

[30] Cárdenas M, Tabima J, Fry WE, Grünwald NJ, Bernal A (2011). Defining species boundaries in the genus *Phytophthora*: The case of *Phytophthora andina*.. Plant Pathology.

[31] Oliva RF, Chacon G, Cooke DEL, Lees AK, Forbes GA (2007). Is *Phytophthora infestans* a good taxonomist? Host recognition and co-evolution in the Phytophthora/Solanum interaction.. Acta Horticulturae.

[32] Gomez-Alpizar L, Hu C-H, Oliva R, Forbes G, Ristaino JB (2008). Phylogenetic relationships of *Phytophthora andina*, a new species from the highlands of Ecuador that is closely related to the Irish potato famine pathogen *Phytophthora infestans*.. Mycologia.

[33] Blair JE, Coffey MD, Park SY, Geiser DM, Kang S (2008). A multi-locus phylogeny for Phytophthora utilizing markers derived from complete genome sequences.. Fungal Genetics and Biology.

[34] Cooke DEL, Drenth A, Duncan JM, Wagels G, Brasier CM (2000). A molecular phylogeny of *Phytophthora* and related oomycetes.. Fungal Genetics and Biology.

[35] Kroon LP, Bakker FT, van den Bosch GB, Bonants PJ, Flier WG (2004). Phylogenetic analysis of Phytophthora species based on mitochondrial and nuclear DNA sequences.. Fungal Genetics and Biology.

[36] Haas BJ, Kamoun S, Zody MC, Jiang RHY, Handsaker RE (2009). Genome sequence and analysis of the Irish potato famine pathogen *Phytophthora infestans*.. Nature.

[37] Raffaele S, Farrer RA, Cano LM, Studholme DJ, MacLean D (2010). Genome evolution following host jumps in the Irish potato famine pathogen lineage.. Science.

[38] Catal M, King L, Tumbalam P, Wiriyajitsomboon P, Kirk WW (2010). Heterokaryotic nuclear conditions and a heterogeneous nuclear population are observed by flow cytometry in *Phytophthora infestans*.. Cytometry Part A.

[39] Tooley PW, Therrien CD (1987). Cytophotometric determination of the nuclear DNA content of 23 Mexican and 18 non-Mexican isolates of *Phytophthora infestans*.. Experimental Mycology.

[40] van der Lee T, Testa A, Robold A, van 't Klooster J, Govers F (2004). High-density genetic linkage maps of *Phytophthora infestans* reveal trisomic progeny and chromosomal rearrangements.. Genetics.

[41] Beser J, Hagblom P, Fernandez V (2007). Frequent in vitro recombination in internal transcribed spacers 1 and 2 during genotyping of *Pneumocystis jirovecii*.. Journal of Clinical Microbiology.

[42] Davey JW, Hohenlohe PA, Etter PD, Boone JQ, Catchen JM (2011). Genome-wide genetic marker discovery and genotyping using next-generation sequencing.. Nature Review Genetics.

[43] Ioos R, Andrieux A, Marcais B, Frey P (2006). Genetic characterization of the natural hybrid species *Phytophthora alni* as inferred from nuclear and mitochondrial DNA analyses.. Fungal Genetics and Biology.

[44] Brasier CM, Kirk SA, Delcan J, Cooke DEL, Jung T (2004). *Phytophthora alni* sp. nov. and its variants: designation of emerging heteroploid hybrid pathogens spreading on *Alnus* trees.. Mycological Research.

[45] Hurtado-Gonzales OP, Aragon-Caballero LM, Flores-Torres JG, Man In'T Veld W, Lamour KH (2009). Molecular comparison of natural hybrids of *Phytophthora nicotianae* and *P. cactorum* infecting loquat trees in Peru and Taiwan.. Mycologia.

[46] Man In'T Veld WA (2001). First report of natural hybrids of *Phytophthora nicotianae* and *P. cactorum* on loquat in Taiwan.. Plant Disease.

[47] Bonants PJM, Hagenaar-de Weerdt M, Man In'T Veld WA, Baayen RP (2000). Molecular characterization of natural hybrids of *Phytophthora nicotianae* and *P. cactorum*.. Phytopathology.

[48] Man In'T Veld WA, de Cock AWAM, Summerbell RC (2007). Natural hybrids of resident and introduced *Phytophthora* species proliferating on multiple new hosts.. European Journal of Plant Pathology.

[49] Man In'T Veld WA, Veenbaas-Rijks WJ, Ilieva E, de Cock AWAM, Bonants PJM (1998). Natural hybrids of *Phytophthora nicotianae* and *P. cactorum* demonstrated by isozyme analysis and random amplified polymorphic DNA.. Phytopathology.

[50] Flier WG, Grünwald NJ, Kroon LPNM, Sturbaum AK, van den Bosch TBM (2003). The population structure of *Phytophthora infestans* from the Toluca Valley of Central Mexico suggests genetic differentiation between populations from cultivated potato and wild *Solanum* spp.. Phytopathology.

[51] Goodwin SB, Legard DE, Smart CD, Levy M, Fry WE (1999). Gene flow analysis of molecular markers confirms that *Phytophthora mirabilis* and *P. infestans* are separate species.. Mycologia.

[52] Kroon LPNM (2010). PhD Thesis: The genus *Phytophthora*; phylogeny, speciation and host specificity.

[53] Oliva Perez RF (2009). PhD Thesis: Occurance of sympatric *Phytophthora* species in the highland of Ecuador.

[54] Coyne JA, Orr HA (2004). Speciation.

[55] Rieseberg LH, Sinervo B, Linder CR, Ungerer MC, Arias DM (1996). Role of gene interactions in hybrid speciation: Evidence from ancient and experimental hybrids.. Science.

[56] Mavarez J, Salazar CA, Bermingham E, Salcedo C, Jiggins CD (2006). Speciation by hybridization in *Heliconius* butterflies.. Nature.

[57] Caten CE, Jinks JL (1968). Spontaneous variability of single isolates of *Phytophthora infestans*. I. Cultural variation.. Canadian Journal of Botany/Revue Canadienne de Botanique.

[58] Griffith GW, Shaw DS (1998). Polymorphisms in *Phytophthora infestans*: Four mitochondrial haplotypes are detected after PCR amplification of DNA from pure cultures or from host lesions.. Applied and Environmental Microbiology.

[59] Chen Y, Roxby R (1996). Characterization of a *Phytophthora infestans* gene involved in vesicle transport.. Gene.

[60] Goss EM, Carbone I, Grünwald NJ (2009). Ancient isolation and independent evolution of the three clonal lineages of the exotic sudden oak death pathogen *Phytophthora ramorum*.. Molecular Ecology.

[61] Tyler BM, Tripathy S, Zhang X, Dehal P, Jiang RH (2006). *Phytophthora* genome sequences uncover evolutionary origins and mechanisms of pathogenesis.. Science.

[62] Cardenas M, Grajales A, Sierra R, Rojas A, Gonzalez-Almario A (2011). Genetic diversity of *Phytophthora infestans* in the Northern Andean region.. BMC Genetics.

[63] Stephens M, Donnelly P (2003). A comparison of bayesian methods for haplotype reconstruction.. American Journal of Human Genetics.

[64] Stephens M, Smith NJ, Donnelly P (2001). A new statistical method for haplotype reconstruction from population data.. American Journal of Human Genetics.

[65] Thompson JD, Higgins DG, Gibson TJ (1994). CLUSTAL W: improving the sensitivity of progressive multiple sequence alignment through sequence weighting, positions-specific gap penalties and weight matrix choice.. Nucleic Acids Research.

[66] Aylor DL, Price EW, Carbone I (2006). SNAP: Combine and Map modules for multilocus population genetic analysis.. Bioinformatics.

[67] Price EW, Carbone I (2005). SNAP: workbench management tool for evolutionary population genetic analysis.. Bioinformatics.

[68] Posada D (2008). jModelTest: Phylogenetic model averaging.. Molecular Biology and Evolution.

[69] Guindon S, Gascuel O (2003). A simple, fast, and accurate algorithm to estimate large phylogenies by maximum likelihood.. Systematic Biology.

[70] Huelsenbeck JP, Ronquist F (2001). MRBAYES: Bayesian inference of phylogenetic trees.. Bioinformatics.

[71] Shimodaira H (2002). An approximately unbiased test of phylogenetic tree selection.. Systematic Biology.

[72] Shimodaira H, Hasegawa M (1999). Multiple comparisons of log-likelihoods with applications to phylogenetic inference.. Molecular Biology and Evolution.

[73] Shimodaira H, Hasegawa M (2001). CONSEL: For assessing the confidence of phylogenetic tree selection.. Bioinformatics.

