# Supplementary figures and images for: The Plant Pathogen Phytophthora andina Emerged via Hybridization of an Unknown Phytophthora Species and the Irish Potato Famine Pathogen, P. infestans
Source: PLoS One. 2011 Sep 16;6(9):e24543. doi: 10.1371/journal.pone.0024543 (PMC3174952; doi:10.1371/journal.pone.0024543)

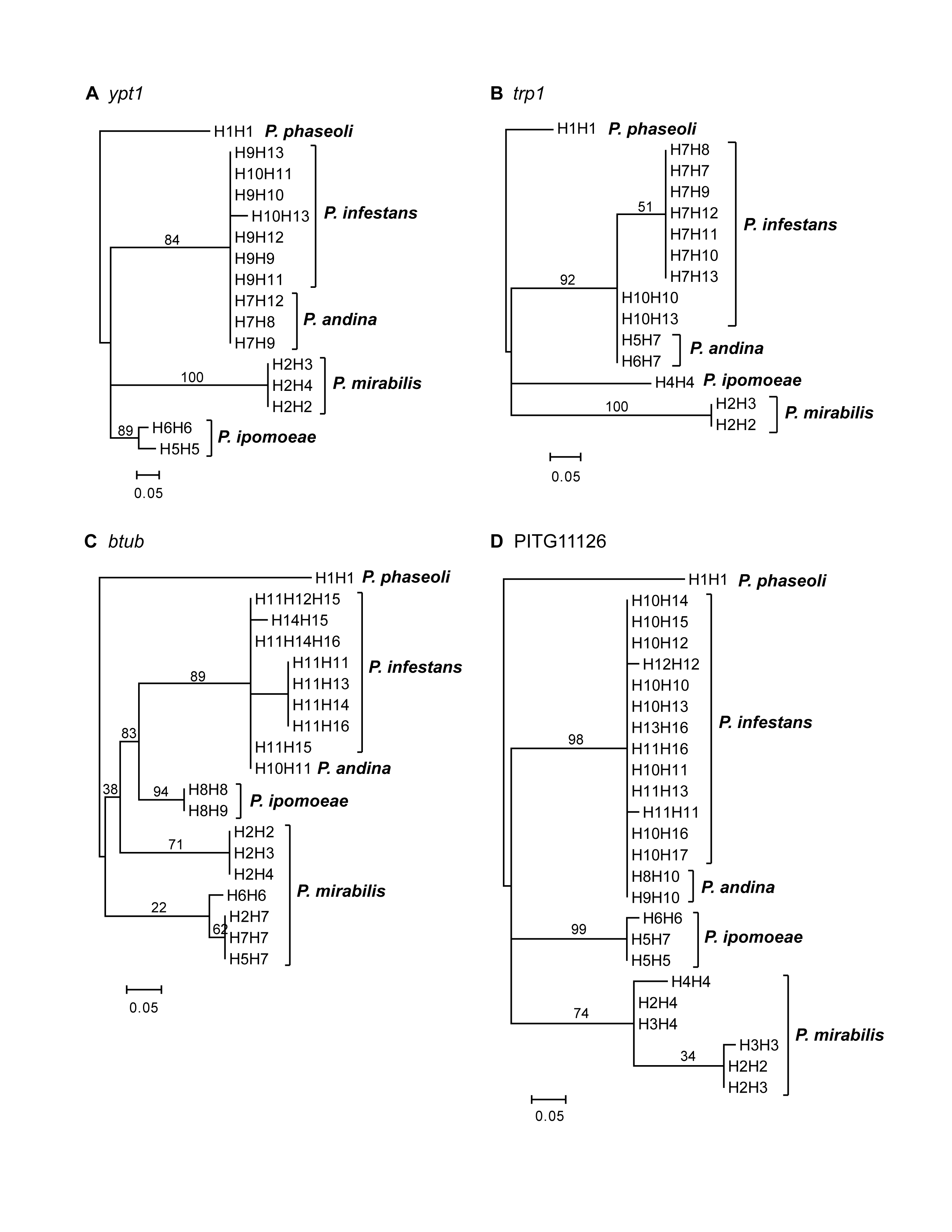

Supplement: Figure S1 — Maximum likelihood trees of genotypes for each locus sequenced in P. andina and four other closely related species. Loci are A. ypt1, B. trp1, C. btub, and D. PITG11126. Genotypes are shown as combinations of haplotypes. Bootstrap support values obtained by maximum likelihood are shown above branches. (TIF) [file pone.0024543.s001.tif]
