# Supplementary material for: The Plant Pathogen Phytophthora andina Emerged via Hybridization of an Unknown Phytophthora Species and the Irish Potato Famine Pathogen, P. infestans
Source: PLoS One. 2011 Sep 16;6(9):e24543. doi: 10.1371/journal.pone.0024543 (PMC3174952; doi:10.1371/journal.pone.0024543)
Supplement: Table S1 — Isolates and haplotype designations for each locus sequenced. (DOCX) [file pone.0024543.s002.docx]

**Table S1**. Isolates and haplotype designations for each locus sequenced.

|  |  |  |  |  | **Haplotype** | | | | |
| --- | --- | --- | --- | --- | --- | --- | --- | --- | --- |
| **Species** | **Isolate** | **Origin** | **Host** | **Source^a^** | **mtDNA^b^** | ***ypt1*** | ***trp1*** | ***btub*** | **PITG11126** |
| *P. andina* | EC 3163 | Ecuador | Anarrichomenum group | CIP | Ic | H7/H9 | H5/H7 | H10/H11 | H8/H10 |
|  | EC 3189 | Ecuador | Anarrichomenum group | CIP | Ic | H7/H10 | H5/H7 | H10/H11 | H8/H10 |
|  | EC 3399 | Ecuador | Anarrichomenum group | CIP | Ia | H7/H10 | H5/H7 | H10/H11 | H8/H10 |
|  | EC 3510 | Ecuador | *S. betaceum* | CIP | Ia | H7/H9 | H6/H7 | H10/H11 | H9/H10 |
|  | EC 3540 | Ecuador | Anarrichomenum group | CIP | Ic | H7/H9 | H5/H7 | H10/H11 | H8/H10 |
|  | EC 3561 | Ecuador | *S. quitoense* | CIP | Ia | H7/H10 | H5/H7 | H10/H11 | H8/H10 |
|  | EC 3563 | Ecuador | *S. quitoense* | CIP | Ia | H7/H10 | H5/H7 | H10/H11 | H8/H10 |
|  | EC 3655 | Ecuador | *S. hispidum* | CIP | Ic | H7/H9 | H5/H7 | H10/H11 | H8/H10 |
|  | EC 3678 | Ecuador | Anarrichomenum group | CIP | Ic | H7/H8 | H5/H7 | H10/H11 | H8/H10 |
|  | EC 3780 | Ecuador | *S. hispidum* | CIP | Ic | H7/H9 | H5/H7 | H10/H11 | H8/H10 |
|  | EC 3818 | Ecuador | Anarrichomenum group | CIP | Ia | H7/H10 | H5/H7 | H10/H11 | H8/H10 |
|  | EC 3821 | Ecuador | Anarrichomenum group | CIP | Ia | H7/H10 | H5/H7 | H10/H11 | H8/H10 |
|  | EC 3836 | Ecuador | *S. betaceum* | CIP | Ia | H7/H9 | H6/H7 | H10/H11 | H9/H10 |
|  | EC 3860 | Ecuador | Torva group | CIP | Ic | H7/H9 | H5/H7 | H10/H11 | H8/H10 |
|  | EC 3864 | Ecuador | Torva group | CIP | Ic | H7/H9 | H5/H7 | H10/H11 | H8/H10 |
|  | POX 102 | Peru | *S. betaceum* | CIP | Ic | H7/H9 | H5/H7 | H10/H11 | H8/H10 |
|  | POX 103 | Peru | *S. betaceum* | CIP | Ic | H7/H9 | H5/H7 | H10/H11 | H8/H10 |
| *P. infestans* | US040009 | USA | Potato | WF | nd | H9/H10 | H7 | H11/H14 | H10 |
|  | US940494 | USA | Tomato | WF | nd | H9/H12 | H7/H9 | H11 | H10/H17 |
|  | US970001 | USA | Tomato | WF | nd | H9/H13 | H7/H12 | H11 | H10/H17 |
|  | US050007 | USA | Tomato | WF | nd | H10/H13 | H7/H13 | H11/H16 | H10/H17 |
|  | MX010006 | Mexico | - | WF | nd | H9/H11 | H7 | H11/H14 | H10 |
|  | MX010046 | Mexico | - | WF | nd | H9/H11 | H7 | H14/H15 | H10/H11 |
|  | MX980211 | Mexico | - | WF | nd | H9/H12 | H7 | H11 | H10 |
|  | MX980230 | Mexico | - | WF | nd | H9/H12 | H7 | H11 | H10 |
|  | MX980317 | Mexico | - | WF | nd | H9 | H7/H10 | H11 | H10/H12 |
|  | MX980352 | Mexico | - | WF | nd | H9/H13 | H7/H12 | H14/H15 | H12 |
|  | MX980400 | Mexico | - | WF | nd | H9 | H7 | H11/H15 | H12 |
|  | PCA 006 | Peru | - | CIP | nd | H9/H10 | H7/H13 | H11 | H11/H13 |
|  | BTLM 004 | Peru | - | CIP | nd | H10/H11 | H7 | H11 | H10/H16 |
|  | PLI 003 | Peru | - | CIP | nd | H10/H11 | H7/H8 | H11 | H10/H16 |
|  | EC 3378 | Ecuador | *S. lycopersicum* | CIP | nd | H9/H11 | H7/H11 | H11 | H10/H14 |
|  | EC 3381 | Ecuador | *S. lycopersicum* | CIP | nd | H9/H11 | H7/H11 | H11 | H10/H14 |
|  | EC 3774 | Ecuador | *S. ochanthum* | CIP | nd | H9/H12 | H7/H13 | H11 | H10 |
|  | 1011 | Colombia | *S. tuberosum* | SR | nd | nd | H7/H13 | H11/H12/H15 | H13/H16 |
|  | 4084 | Colombia | *Physalis peruviana* | SR | nd | H9/H10 | H7 | H11/H14/H16 | H10/H17 |
|  | 2004 4 | Estonia | Potato | WF | nd | H9/H10 | H10 | H11 | H10/H11 |
|  | 2004 16 | Estonia | Potato | WF | nd | H9/H10 | H10/H13 | H11/H13 | H10/H13 |
|  | 2006 3984C | UK | Potato | DC | nd | H10/H11 | H7/H13 | H11/H13 | H11/H16 |
|  | 2006 4388D | UK | Potato | DC | nd | H9/H10 | H7/H13 | H11 | H11 |
|  | Vn02-076 | Vietnam | Tomato | AH | nd | H9/H11 | H7/H11 | H11 | H10/H15 |
|  | Vn03-590 | Vietnam | Potato | AH | nd | H9/H11 | H7/H11 | H11 | H10/H15 |
| *P. ipomoeae* | Ipom2-1 | Mexico | *Ipomoea longipedunculata* | PRI | - | H5 | H4 | H8 | H5 |
|  | Ipom3-3 | Mexico | *Ipomoea longipedunculata* | PRI | - | H5 | H4 | H8 | H5 |
|  | Ipom6 | Mexico | *Ipomoea longipedunculata* | PRI | - | H5 | H4 | H8 | H5 |
|  | Ipom2-4 | Mexico | *Ipomoea longipedunculata* | PRI | - | H5 | H4 | H8 | H5 |
|  | Ipom1-2 | Mexico | *Ipomoea longipedunculata* | PRI | - | H5 | H4 | H8 | H5 |
|  | 00Ip5 | Mexico | *Ipomoea longipedunculata* | PRI | - | H5 | H4 | H8 | H5/H7 |
|  | Pi Mich | Mexico | *Ipomoea longipedunculata* | PRI | - | H6 | H4 | H8/H9 | H6 |
| *P. mirabilis* | CBS 678.85 | Mexico | *Mirabilis jalapa* | CBS | - | nd | H2 | H6 | H2/H3 |
|  | CBS 136.86 | Mexico | *Mirabilis jalapa* | CBS | - | H2 | H2 | H5/H7 | H3/H4 |
|  | P 3001 | Mexico | *Mirabilis jalapa* | PRI | - | H2 | H2 | H5/H7 | H3/H4 |
|  | P 3006 | Mexico | *Mirabilis jalapa* | PRI | - | H2 | H2 | H2/H4 | H3/H4 |
|  | G 11-3 | Mexico | *Mirabilis jalapa* | PRI | - | H2 | H2 | H2/H3 | H2 |
|  | PIC99114 | Mexico | *Mirabilis jalapa* | PRI | - | H2/H3 | H2 | H2/H3 | H2 |
|  | PIC99135 | Mexico | *Mirabilis jalapa* | PRI | - | H2/H4 | H2 | H2/H3 | H2/H3 |
|  | DF 409 | Mexico | *Mirabilis jalapa* | PRI | - | H2 | H2 | H2/H7 | H2/H4 |
|  | 00M 410 | Mexico | *Mirabilis jalapa* | PRI | - | H2 | H2 | H2 | H4 |
|  | Pm Mich | Mexico | *Mirabilis jalapa* | PRI | - | H2 | H2/H3 | H7 | H2 |
|  | DF 07 | Mexico | *Mirabilis jalapa* | PRI | - | H2 | H2 | H2 | H3 |
| *P. phaseoli* | CBS556.88 | - | *Phaseolus lunatus* | CBS | - | H1 | H1 | H1 | H1 |

^a^ AH – Arne Hermansen, The Norwegian Crop Research Institute; CBS – Centraalbureau voor Schimmelcultures, The Netherlands; CIP –International Potato Center; DC – David Cooke, Scottish Crop Research Institute; PRI –Plant Research International; SR – Silvia Restrepo, Universidad de los Andes; WF – William Fry, Cornell University.

^b^ Haplotype designations for *P. andina* as described in references [[28](#_ENREF_28),[58](#_ENREF_58)]. nd – mtDNA haplotypes were not determined for the other species.
