# Supplementary material for: The Plant Pathogen Phytophthora andina Emerged via Hybridization of an Unknown Phytophthora Species and the Irish Potato Famine Pathogen, P. infestans
Source: PLoS One. 2011 Sep 16;6(9):e24543. doi: 10.1371/journal.pone.0024543 (PMC3174952; doi:10.1371/journal.pone.0024543)
Supplement: Table S2 — Variable sites at each sequenced locus: A. ypt1 , B. trp1 , C. btub , and D. PITG11126. For each locus, the consensus sequence across clade 1c species is shown and identity to this sequence indicated with a dot. Haplotype numbers for each locus correspond with those in Tables 1, 2, and S1, and Figure 2. Site numbers indicate position in multispecies alignment. Indels are not included; see Tables S3, S4, S5, S6, S7, S8 for indels that are heterozygous in P. andina. Sites with shared nucleotides between the non-P. infestans haplotype in P. andina and P. mirabilis, P. ipomoeae, or P. infestans are shown in bold. (DOCX) [file pone.0024543.s003.docx]

**Table S2**. Variable sites at each sequenced locus: **A**. *ypt1*, **B**. *trp1*, **C**. *btub*, and **D**. PITG11126. For each locus, the consensus sequence across clade 1c species is shown and identity to this sequence indicated with a dot. Haplotype numbers for each locus correspond with those in Tables 1, 2, and S1, and Figure 2. Site numbers indicate position in multispecies alignment. Indels are not included, see Tables S3 through S8 for indels that are heterozygous in *P. andina*. Sites with shared nucleotides between the non-*P. infestans* haplotype in *P. andina* and *P. mirabilis*, *P. ipomoeae*, or *P. infestans* are shown in bold.

**A. *ypt1***

| Site |  |  |  |  |  |  |  |  |  |  |  | 1 | 1 | 1 | 1 | **1** | 2 | 2 | 2 | 2 | 4 | **5** | **5** | 5 | 5 | 5 | 5 | 5 | 5 | 6 | 6 | 6 | 6 | 6 | **6** | 6 | 6 | 6 | 6 | 6 | 6 | 6 | 6 | 6 | 7 | 7 | 7 | 8 | 8 | 8 | 8 | 8 | 8 | **8** | **8** | **8** |
| --- | --- | --- | --- | --- | --- | --- | --- | --- | --- | --- | --- | --- | --- | --- | --- | --- | --- | --- | --- | --- | --- | --- | --- | --- | --- | --- | --- | --- | --- | --- | --- | --- | --- | --- | --- | --- | --- | --- | --- | --- | --- | --- | --- | --- | --- | --- | --- | --- | --- | --- | --- | --- | --- | --- | --- | --- |
|  |  |  | 2 | 2 | 5 | 6 | 7 | 8 | 9 | 9 | 9 | 0 | 0 | 1 | 1 | **9** | 0 | 1 | 1 | 1 | 9 | **1** | **1** | 3 | 5 | 6 | 7 | 7 | 8 | 3 | 4 | 5 | 5 | 5 | **5** | 5 | 7 | 8 | 8 | 8 | 9 | 9 | 9 | 9 | 2 | 4 | 5 | 1 | 5 | 5 | 5 | 6 | 6 | **7** | **7** | **8** |
|  |  | 1 | 1 | 9 | 3 | 3 | 1 | 4 | 1 | 5 | 8 | 0 | 5 | 2 | 6 | **3** | 6 | 1 | 2 | 4 | 4 | **2** | **9** | 3 | 7 | 9 | 3 | 9 | 0 | 6 | 9 | 0 | 2 | 4 | **8** | 9 | 8 | 4 | 5 | 6 | 0 | 2 | 3 | 8 | 0 | 7 | 6 | 0 | 0 | 7 | 8 | 2 | 7 | **0** | **1** | **0** |
|  |  |  |  |  |  |  |  |  |  |  |  |  |  |  |  |  |  |  |  |  |  |  |  |  |  |  |  |  |  |  |  |  |  |  |  |  |  |  |  |  |  |  |  |  |  |  |  |  |  |  |  |  |  |  |  |  |
| Consensus |  | G | G | A | T | C | C | C | A | G | G | C | C | G | G | **C** | G | G | C | G | G | **A** | **A** | G | G | G | T | C | C | C | C | A | T | G | **C** | T | C | G | T | T | C | A | T | A | G | T | C | C | C | T | T | G | G | **C** | **C** | **A** |
| Species |  |  |  |  |  |  |  |  |  |  |  |  |  |  |  |  |  |  |  |  |  |  |  |  |  |  |  |  |  |  |  |  |  |  |  |  |  |  |  |  |  |  |  |  |  |  |  |  |  |  |  |  |  |  |  |  |
| *P. phaseoli* | H1 | T | . | . | . | . | T | . | . | . | . | . | . | C | . | **.** | . | . | . | . | A | **.** | **.** | . | . | . | . | . | . | . | . | . | G | . | **T** | . | . | A | C | . | G | . | . | . | . | . | A | . | . | C | . | A | . | **.** | **.** | **.** |
| *P. mirabilis* | H2 | . | . | . | . | . | . | T | . | . | . | . | . | . | . | **.** | . | . | . | . | . | **G** | **G** | . | . | . | . | . | . | T | . | . | . | . | **.** | . | . | . | . | A | T | T | . | T | . | . | . | . | . | . | . | . | C | **T** | **T** | **T** |
|  | H3 | . | . | G | . | . | . | . | . | . | . | . | . | . | . | **.** | . | . | . | . | . | **G** | **G** | . | . | . | A | . | . | T | A | T | . | . | **.** | . | T | . | . | A | T | T | . | T | . | . | . | . | . | . | . | . | C | **T** | **T** | **T** |
|  | H4 | . | . | . | . | . | . | T | . | . | . | . | . | . | . | **.** | . | . | . | . | . | **G** | **G** | . | . | . | . | . | . | T | . | G | . | . | **.** | . | . | . | . | A | T | T | . | T | . | . | . | . | . | . | . | . | C | **T** | **T** | **T** |
| *P. ipomoeae* | H5 | . | . | . | . | . | . | . | . | . | . | . | . | . | . | **T** | . | . | T | A | . | **.** | **.** | . | . | . | . | . | . | . | . | . | . | T | **T** | . | . | . | C | . | . | . | . | . | . | . | . | . | T | . | . | . | . | **.** | **.** | **.** |
|  | H6 | . | . | . | . | . | . | . | . | . | . | . | . | . | T | **T** | . | . | . | . | . | **.** | **.** | . | . | . | . | . | . | . | . | . | . | T | **T** | . | . | . | C | . | . | . | . | . | . | . | . | . | T | . | . | . | . | **.** | **.** | **.** |
| *P. andina* | H7 | . | A | . | . | . | . | . | . | . | . | . | . | . | . | **T** | . | A | . | . | . | **G** | **G** | . | . | . | . | . | . | . | . | . | . | . | **T** | . | . | . | . | . | . | . | . | . | . | . | . | . | . | . | . | . | . | **T** | **T** | **T** |
|  | H8 | . | . | . | C | . | . | . | C | A | . | A | A | A | . | **.** | . | . | . | . | . | **.** | **.** | . | C | . | . | T | T | . | . | . | . | . | **.** | C | . | . | . | . | . | . | . | . | . | C | . | T | . | . | . | . | . | **.** | **.** | **.** |
| *P. inf.* & *P. and.* | H9 | . | . | . | C | . | . | . | C | A | . | A | A | A | . | **.** | . | . | . | . | . | **.** | **.** | . | C | . | . | T | T | . | . | . | . | . | **.** | C | . | . | . | . | . | . | . | . | . | . | . | T | . | . | . | . | . | **.** | **.** | **.** |
|  | H10 | . | . | . | C | . | . | . | C | A | . | A | A | A | . | **.** | . | . | . | . | . | **.** | **.** | . | C | A | . | T | T | . | . | . | . | . | **.** | C | . | . | . | . | . | . | C | . | . | . | . | T | . | . | . | . | . | **.** | **.** | **.** |
| *P. infestans* | H11 | . | . | . | C | T | . | . | C | A | T | A | . | . | . | **.** | A | . | . | . | . | **.** | **.** | . | C | . | . | T | T | . | . | . | . | . | **.** | C | . | . | . | . | . | . | . | . | A | . | . | . | . | . | A | . | . | **.** | **.** | **.** |
|  | H12 | . | . | . | C | . | . | . | C | A | . | A | A | A | . | **.** | . | . | . | . | . | **.** | **.** | . | C | A | . | T | T | . | . | . | . | . | **.** | C | . | . | . | . | . | . | . | . | . | . | . | T | . | . | . | . | . | **.** | **.** | **.** |
|  | H13 | . | . | . | C | . | . | . | C | A | . | A | A | A | . | **.** | . | . | . | . | . | **.** | **.** | T | C | A | . | T | T | . | . | . | . | . | **.** | C | . | . | . | . | . | . | C | . | . | . | . | T | . | . | . | . | . | **.** | **.** | **.** |
|  | H14 | . | . | . | C | . | . | . | C | A | . | A | A | A | . | **.** | . | . | . | . | . | **.** | **.** | . | C | A | . | T | T | . | . | . | . | . | **.** | C | . | T | . | . | . | . | . | . | . | . | . | T | . | . | . | . | . | **.** | **.** | **.** |

**B. *trp1***

| Site |  |  |  |  |  |  | 1 | 1 | 1 | **1** | 1 | 1 | 2 | 2 | 2 | 3 | 3 | 4 | 5 | 5 | 6 | 6 | 6 | 7 | 7 | 7 | 7 |
| --- | --- | --- | --- | --- | --- | --- | --- | --- | --- | --- | --- | --- | --- | --- | --- | --- | --- | --- | --- | --- | --- | --- | --- | --- | --- | --- | --- |
|  |  | 2 | 3 | 6 | 6 | 8 | 2 | 4 | 4 | **6** | 8 | 8 | 3 | 8 | 9 | 2 | 9 | 1 | 4 | 5 | 1 | 3 | 6 | 2 | 5 | 8 | 8 |
|  |  | 6 | 9 | 3 | 6 | 9 | 0 | 3 | 7 | **9** | 0 | 1 | 6 | 0 | 9 | 9 | 8 | 3 | 7 | 2 | 2 | 7 | 9 | 9 | 4 | 0 | 3 |
|  |  |  |  |  |  |  |  |  |  |  |  |  |  |  |  |  |  |  |  |  |  |  |  |  |  |  |  |
| Consensus |  | G | G | T | G | T | A | C | C | **T** | T | A | T | G | C | G | C | C | G | A | C | C | C | C | C | T | G |
| Species |  |  |  |  |  |  |  |  |  |  |  |  |  |  |  |  |  |  |  |  |  |  |  |  |  |  |  |
| *P. phaseoli* | H1 | . | . | . | . | . | . | . | . | **.** | . | . | . | . | . | . | T | . | . | . | . | . | A | . | . | . | . |
| *P. mirabilis* | H2 | A | . | . | . | . | . | . | . | **A** | A | . | G | T | . | . | . | . | . | . | . | . | . | . | . | C | . |
|  | H3 | A | . | . | . | . | . | . | . | **A** | A | . | G | T | . | . | . | . | . | . | . | T | . | . | . | C | . |
| *P. ipomoeae* | H4 | . | . | . | . | . | . | T | T | **.** | . | . | . | . | . | . | . | . | . | . | T | . | . | . | T | . | C |
| *P. andina* | H5 | . | . | . | . | . | . | . | . | **A** | . | C | . | . | T | . | . | . | A | . | . | . | . | . | . | . | . |
|  | H6 | . | . | . | . | . | . | . | . | **A** | . | C | . | . | . | . | . | . | A | . | . | . | . | . | . | . | . |
| *P. inf.* & *P. and.* | H7 | . | . | . | A | . | G | . | . | **.** | . | . | . | . | . | A | . | T | . | G | . | . | . | T | . | . | . |
| *P. infestans* | H8 | . | . | . | A | A | G | . | . | **.** | . | . | . | . | . | A | . | T | . | G | . | . | . | T | . | . | . |
|  | H9 | . | . | . | A | . | . | . | . | **.** | . | . | . | . | . | A | . | T | . | G | . | . | . | T | . | . | . |
|  | H10 | . | . | . | A | . | . | . | . | **.** | . | . | . | . | . | A | . | T | . | . | . | . | . | T | . | . | . |
|  | H11 | . | A | . | A | . | . | . | . | **.** | . | . | . | . | . | A | . | T | . | . | . | . | . | T | . | . | . |
|  | H12 | . | . | G | A | . | . | . | . | **.** | . | . | . | . | . | A | . | T | . | . | . | . | . | T | . | . | . |
|  | H13 | . | . | . | . | . | . | . | . | **.** | . | . | . | . | . | A | . | T | . | . | . | . | . | T | . | . | . |

**C. *btub***

| Site |  |  |  |  |  |  |  |  |  |  |  |  |  |  |  |  |  |  |  |  |  |  |  |  |  |  |  |  |  |  |  |  |  |  |  |  |  | 1 | 1 | 1 | 1 | **1** | **1** | 1 | 1 | 1 | 1 | 1 | **1** | 1 | 1 | 1 |
| --- | --- | --- | --- | --- | --- | --- | --- | --- | --- | --- | --- | --- | --- | --- | --- | --- | --- | --- | --- | --- | --- | --- | --- | --- | --- | --- | --- | --- | --- | --- | --- | --- | --- | --- | --- | --- | --- | --- | --- | --- | --- | --- | --- | --- | --- | --- | --- | --- | --- | --- | --- | --- |
|  |  |  |  |  |  |  |  |  |  |  | 1 | 1 | 1 | 1 | 1 | 1 | 2 | 3 | 3 | 3 | 3 | 3 | 4 | 4 | 5 | **5** | **6** | 6 | 6 | 7 | 7 | 7 | 8 | **8** | 9 | **9** | 9 | 0 | 0 | 1 | 1 | **1** | **1** | 1 | 2 | 2 | 3 | 3 | **4** | 4 | 4 | 5 |
|  |  | 3 | 4 | 4 | 5 | 6 | 7 | 7 | 7 | 9 | 3 | 6 | 6 | 6 | 8 | 9 | 9 | 3 | 3 | 5 | 8 | 8 | 1 | 5 | 3 | **4** | **1** | 3 | 6 | 1 | 2 | 3 | 1 | **5** | 0 | **6** | 6 | 2 | 8 | 3 | 4 | **5** | **5** | 5 | 0 | 7 | 5 | 8 | **4** | 4 | 5 | 7 |
|  |  | 0 | 1 | 4 | 0 | 4 | 0 | 3 | 6 | 3 | 9 | 4 | 5 | 7 | 5 | 6 | 4 | 3 | 6 | 7 | 1 | 7 | 7 | 3 | 4 | **3** | **2** | 6 | 9 | 7 | 3 | 8 | 0 | **2** | 0 | **1** | 6 | 6 | 0 | 4 | 9 | **2** | **5** | 8 | 0 | 8 | 9 | 0 | **0** | 6 | 2 | 1 |
|  |  |  |  |  |  |  |  |  |  |  |  |  |  |  |  |  |  |  |  |  |  |  |  |  |  |  |  |  |  |  |  |  |  |  |  |  |  |  |  |  |  |  |  |  |  |  |  |  |  |  |  |  |
| Consensus |  | T | T | C | C | T | T | C | A | T | G | G | T | G | G | C | G | G | C | T | C | T | C | C | T | **T** | **C** | C | C | G | C | G | C | **C** | G | **C** | T | T | G | C | T | **A** | **T** | T | G | C | C | C | **C** | T | C | G |
| Species |  |  |  |  |  |  |  |  |  |  |  |  |  |  |  |  |  |  |  |  |  |  |  |  |  |  |  |  |  |  |  |  |  |  |  |  |  |  |  |  |  |  |  |  |  |  |  |  |  |  |  |  |
| *P. phaseoli* | H1 | . | . | . | . | C | . | G | . | . | . | T | G | . | . | . | . | . | . | . | . | C | T | . | G | **.** | **.** | . | . | . | . | . | . | **.** | T | **.** | C | . | . | . | A | **C** | **G** | . | . | . | . | . | **.** | . | . | . |
| *P. mirabilis* | H2 | C | . | . | A | C | . | . | . | C | . | . | . | . | . | . | . | . | . | . | . | . | . | G | . | **.** | **.** | . | . | . | T | . | . | **.** | . | **.** | . | . | . | . | . | **C** | **.** | . | . | . | . | . | **.** | . | . | . |
|  | H3 | C | . | . | A | C | . | . | . | C | . | . | . | . | . | . | . | . | . | . | . | . | . | G | . | **.** | **.** | . | . | . | T | . | . | **.** | . | **.** | . | . | . | . | . | **C** | **.** | . | . | . | T | . | **.** | . | . | . |
|  | H4 | . | . | . | . | . | . | . | . | . | . | . | . | . | . | . | . | . | T | . | . | . | . | . | . | **C** | **.** | . | . | . | T | . | . | **.** | . | **.** | . | . | . | . | . | **C** | **.** | . | . | . | . | . | **.** | . | . | . |
|  | H5 | . | . | . | . | . | . | . | . | . | . | . | . | . | . | . | . | . | T | . | . | . | . | . | . | **C** | **T** | . | . | . | . | . | . | **T** | . | **T** | . | . | . | . | . | **C** | **G** | . | A | . | . | . | **.** | . | . | . |
|  | H6 | . | . | . | . | . | . | . | . | . | . | . | . | . | . | . | . | . | T | C | . | . | . | . | . | **C** | **T** | . | . | . | . | . | . | **T** | . | **T** | . | . | . | . | . | **C** | **G** | . | A | . | . | . | **.** | . | . | . |
|  | H7 | . | . | . | . | . | . | . | . | . | . | . | . | . | . | . | . | . | T | . | . | . | . | . | . | **C** | **T** | . | . | T | . | . | . | **T** | . | **T** | . | . | . | . | . | **C** | **G** | . | A | . | . | . | **.** | . | . | . |
| *P. ipomoeae* | H8 | . | . | . | . | . | . | . | . | . | . | . | . | . | . | . | . | . | . | . | . | . | . | . | . | **.** | **.** | . | . | . | . | . | T | **.** | . | **.** | . | . | . | . | . | **.** | **.** | . | . | T | . | . | **.** | C | . | . |
|  | H9 | . | . | . | . | . | . | . | . | . | . | . | . | . | . | . | . | . | . | . | . | . | . | . | . | **.** | **.** | . | . | . | . | . | T | **.** | . | **.** | . | . | . | . | . | **.** | **.** | . | . | T | . | . | **.** | . | T | . |
| *P. andina* | H10 | . | C | . | . | . | . | . | . | . | . | . | . | . | . | . | A | . | . | . | T | . | . | . | . | **C** | **T** | T | T | . | . | . | . | **T** | . | **T** | . | C | A | . | . | **C** | **G** | . | . | . | . | . | **T** | . | . | T |
| *P. inf.* & *P. and.* | H11 | . | . | . | . | . | G | . | G | . | A | . | . | A | . | T | . | A | . | . | . | . | T | . | . | **.** | **.** | . | . | . | . | A | . | **.** | . | **.** | . | . | . | . | . | **.** | **.** | . | . | . | . | T | **T** | . | . | . |
| *P. infestans* | H12 | . | . | . | . | . | G | . | G | . | A | . | . | A | . | T | . | A | . | . | . | . | T | . | . | **.** | **.** | . | . | . | . | A | . | **.** | . | **.** | . | . | . | T | . | **.** | **.** | C | . | . | . | . | **T** | . | . | . |
|  | H13 | . | . | . | . | . | G | . | G | . | A | . | . | A | . | T | . | A | . | . | . | . | T | . | . | **.** | **.** | . | . | . | . | A | . | **.** | . | **.** | . | . | . | T | . | **.** | **.** | . | . | . | . | . | **T** | . | . | . |
|  | H14 | . | . | T | . | . | G | . | G | . | . | . | . | A | . | . | . | A | . | . | . | . | T | . | . | **.** | **.** | . | . | . | . | A | . | **.** | . | **.** | . | . | . | T | . | **.** | **.** | . | . | . | . | . | **T** | . | . | . |
|  | H15 | . | . | . | . | . | A | . | G | . | . | . | . | A | T | . | . | A | . | . | . | . | T | . | . | **.** | **.** | . | . | . | . | A | . | **.** | . | **.** | . | . | . | T | . | **.** | **.** | . | . | . | . | . | **T** | . | . | . |
|  | H16 | . | . | . | . | . | G | . | G | . | . | . | . | A | . | . | . | A | . | . | . | . | T | . | . | **.** | **.** | . | . | . | . | A | . | **.** | . | **.** | . | . | . | T | . | **.** | **.** | . | . | . | . | . | **T** | . | . | . |

**D. PITG11126**

| Site |  |  |  |  |  |  |  |  |  |  |  | 1 | 1 | **1** | 1 | 1 | 1 | 1 | 1 | 2 | 2 | **2** | 2 | 2 | 2 | 2 | **2** | 3 | 3 | 3 | 3 | 3 | 3 | 3 | 4 | 4 | 4 | 4 | 4 | 4 | 4 | 5 | 5 | 5 | 5 | 5 | 5 | 5 | 5 | 5 | 6 | 6 | **6** | 6 | 6 | 6 | 6 | 7 | **7** | **7** | 7 | 7 |
| --- | --- | --- | --- | --- | --- | --- | --- | --- | --- | --- | --- | --- | --- | --- | --- | --- | --- | --- | --- | --- | --- | --- | --- | --- | --- | --- | --- | --- | --- | --- | --- | --- | --- | --- | --- | --- | --- | --- | --- | --- | --- | --- | --- | --- | --- | --- | --- | --- | --- | --- | --- | --- | --- | --- | --- | --- | --- | --- | --- | --- | --- | --- |
|  |  |  | 4 | 4 | 5 | **6** | 6 | 6 | 7 | 7 | 7 | 1 | 2 | **2** | 3 | 4 | 5 | 7 | 7 | 0 | 3 | **5** | 5 | 5 | 8 | 8 | **8** | 0 | 0 | 1 | 1 | 6 | 6 | 7 | 0 | 0 | 2 | 3 | 4 | 5 | 9 | 0 | 1 | 2 | 2 | 2 | 4 | 4 | 8 | 8 | 1 | 1 | **1** | 2 | 3 | 5 | 7 | 2 | **3** | **3** | 5 | 5 |
|  |  | **9** | 1 | 8 | 5 | **1** | 5 | 9 | 1 | 5 | 6 | 2 | 3 | **4** | 1 | 4 | 9 | 3 | 6 | 1 | 9 | **2** | 8 | 9 | 2 | 4 | **8** | 3 | 4 | 3 | 8 | 3 | 6 | 6 | 2 | 4 | 8 | 5 | 4 | 2 | 4 | 5 | 9 | 1 | 2 | 3 | 1 | 6 | 4 | 7 | 2 | 3 | **4** | 6 | 7 | 8 | 3 | 6 | **7** | **8** | 2 | 9 |
|  |  |  |  |  |  |  |  |  |  |  |  |  |  |  |  |  |  |  |  |  |  |  |  |  |  |  |  |  |  |  |  |  |  |  |  |  |  |  |  |  |  |  |  |  |  |  |  |  |  |  |  |  |  |  |  |  |  |  |  |  |  |  |
| Consensus |  | **G** | C | C | C | **T** | C | C | A | G | G | C | G | **C** | G | C | G | A | C | G | G | **G** | A | T | T | A | **T** | A | C | C | A | T | G | C | A | T | C | A | C | G | C | G | A | T | T | C | C | C | C | C | A | G | **T** | C | T | C | C | C | **G** | **C** | C | A |
| Species |  |  |  |  |  |  |  |  |  |  |  |  |  |  |  |  |  |  |  |  |  |  |  |  |  |  |  |  |  |  |  |  |  |  |  |  |  |  |  |  |  |  |  |  |  |  |  |  |  |  |  |  |  |  |  |  |  |  |  |  |  |  |
| *P. phaseoli* | H1 | **.** | . | T | T | **.** | . | . | T | A | A | . | . | **.** | . | . | . | . | T | . | . | **.** | . | . | . | . | **.** | . | . | T | . | . | . | . | . | C | . | . | . | . | G | . | . | . | . | . | . | . | T | T | . | A | **G** | . | . | . | . | . | **.** | **.** | T | . |
| *P. mirabilis* | H2 | **.** | . | . | . | **.** | . | T | . | . | . | A | . | **T** | . | . | . | . | . | A | . | **.** | . | C | . | . | **.** | T | T | T | . | . | . | . | . | . | . | . | T | . | . | . | . | C | A | G | . | . | . | T | . | A | **G** | . | . | . | A | . | **.** | **.** | . | . |
|  | H3 | **.** | . | . | . | **.** | . | T | . | . | . | A | . | **T** | . | . | . | . | . | A | . | **.** | . | C | . | G | **.** | T | T | T | . | . | . | . | . | . | . | . | T | . | . | . | . | C | A | G | . | . | . | T | . | A | **G** | . | . | . | A | . | **.** | **.** | . | . |
|  | H4 | **.** | . | . | . | **.** | . | T | . | . | . | A | . | **T** | . | . | . | . | . | A | . | **.** | . | C | . | . | **.** | T | T | T | . | G | A | . | . | . | . | . | T | . | . | . | . | . | . | . | . | . | . | . | C | . | **.** | . | . | . | . | . | **.** | **.** | . | . |
| *P. ipomoeae* | H5 | **.** | . | . | . | **A** | T | . | . | . | . | . | A | **.** | A | . | . | . | . | . | A | **.** | . | . | . | . | **.** | . | . | . | . | . | . | . | G | . | . | . | . | . | . | . | . | . | . | . | . | . | . | . | . | . | **.** | . | C | . | . | . | **.** | **.** | A | . |
|  | H6 | **.** | . | . | . | **A** | T | . | . | . | . | . | A | **.** | A | . | . | . | . | . | A | **.** | . | . | . | . | **.** | . | . | . | . | . | . | . | G | . | . | . | . | . | . | . | . | . | . | . | . | . | . | . | . | . | **.** | . | C | A | . | . | **.** | **.** | A | . |
|  | H7 | **.** | . | . | . | **A** | T | . | . | . | . | . | A | **.** | A | . | . | . | . | . | A | **.** | . | . | . | . | **.** | . | . | . | . | . | . | . | G | . | . | . | . | . | . | . | . | . | . | . | . | . | . | . | . | . | **.** | T | C | . | . | . | **.** | **.** | A | . |
| *P. andina* | H8 | **A** | . | . | . | **A** | . | . | . | . | . | . | . | **T** | . | . | . | G | . | . | . | **T** | . | . | . | . | **C** | . | . | . | . | . | . | . | . | . | T | G | . | . | . | A | . | . | . | T | . | . | . | . | T | C | **G** | . | . | . | . | . | **A** | **T** | . | . |
|  | H9 | **A** | . | . | . | **A** | . | . | . | . | . | . | . | **T** | . | . | . | G | . | . | . | **T** | . | . | . | . | **C** | . | . | . | . | . | . | . | . | . | T | G | . | . | . | . | T | . | . | T | . | . | . | . | . | . | **.** | . | . | . | . | . | **A** | **T** | . | . |
| *P. inf.* & *P. and.* | H10 | **A** | . | . | . | **.** | . | . | . | . | . | . | . | **.** | . | . | . | . | . | . | . | **T** | G | . | . | . | **.** | . | . | . | . | . | . | T | . | . | . | . | . | . | . | . | . | . | . | . | T | G | . | . | . | . | **.** | . | . | . | . | . | **A** | **T** | . | . |
| *P. infestans* | H11 | **A** | . | . | . | **.** | . | . | . | . | . | . | . | **.** | . | . | . | . | . | . | . | **T** | G | . | . | . | **C** | . | . | . | . | . | . | T | . | . | . | . | . | . | . | . | . | . | . | . | T | G | . | . | . | . | **.** | . | . | . | . | . | **A** | **T** | . | . |
|  | H12 | **A** | . | . | . | **.** | . | . | . | . | . | . | . | **.** | . | . | . | . | . | . | . | **T** | G | . | A | . | **.** | . | . | . | . | . | . | T | . | . | . | . | . | . | . | . | . | . | . | . | T | G | . | . | . | . | **.** | . | . | . | . | . | **A** | **T** | . | . |
|  | H13 | **A** | . | . | . | **.** | . | . | . | . | . | . | . | **.** | . | . | A | . | . | . | . | **T** | G | . | . | . | **.** | . | . | . | . | . | . | T | . | . | . | . | . | . | . | . | . | . | . | . | T | G | . | . | . | . | **.** | . | . | . | . | . | **A** | **T** | . | . |
|  | H14 | **A** | T | . | . | **.** | . | . | . | . | . | . | . | **.** | . | T | . | . | . | . | . | **T** | G | . | . | . | **.** | . | . | . | . | . | . | T | . | . | . | . | . | A | . | . | . | . | . | . | T | G | . | . | . | . | **.** | . | . | . | . | T | **A** | **T** | . | . |
|  | H15 | **A** | T | . | . | **.** | . | . | . | . | . | . | . | **.** | . | . | . | . | . | . | . | **T** | G | . | . | . | **.** | . | . | . | . | . | . | T | . | . | . | . | . | A | . | . | . | . | . | . | T | G | . | . | . | . | **.** | . | . | . | . | T | **A** | **T** | . | . |
|  | H16 | **A** | . | . | . | **.** | . | . | . | . | . | . | . | **.** | . | . | . | . | . | . | . | **T** | G | . | . | . | **.** | . | . | . | G | . | . | T | . | . | . | . | . | . | . | . | . | . | . | . | . | A | . | . | . | . | **.** | . | . | . | . | T | **A** | **T** | . | G |
|  | H17 | **A** | . | . | . | **.** | . | . | . | . | . | . | . | **.** | . | . | . | . | . | . | . | **T** | G | . | . | . | **.** | . | . | . | . | . | . | T | . | . | . | . | . | . | . | . | . | . | . | . | . | A | . | . | . | . | **.** | . | . | . | . | . | **A** | **T** | . | . |
