# Supplementary material for: The Plant Pathogen Phytophthora andina Emerged via Hybridization of an Unknown Phytophthora Species and the Irish Potato Famine Pathogen, P. infestans
Source: PLoS One. 2011 Sep 16;6(9):e24543. doi: 10.1371/journal.pone.0024543 (PMC3174952; doi:10.1371/journal.pone.0024543)
Supplement: Table S3 — P. andina ypt1 haplotypes obtained from cloning for the full region (IR through RAS). Italicized sites are between sequenced regions and were not included in the analysis. (DOCX) [file pone.0024543.s004.docx]

**Table S3.** *P. andina ypt1* haplotypes obtained from cloning for the full region (IR through RAS). Italicized sites are between sequenced regions and were not included in the analysis.

| Site |  |  |  | 21 | 53 | 91 | 95 | 100 | 105 | 112 | 183 | 184 | 185 | 193 | 211 | *240* | *244* | *328* | *346* | *353* | *410* | *431* | 512 | 519 | 557 | 569 | 579 | 580 | 657 | 658 | 664 | 692 | 808 | 868 | 869 | 878 |
| --- | --- | --- | --- | --- | --- | --- | --- | --- | --- | --- | --- | --- | --- | --- | --- | --- | --- | --- | --- | --- | --- | --- | --- | --- | --- | --- | --- | --- | --- | --- | --- | --- | --- | --- | --- | --- |
| Isolate | IR^a^ | RAS^b^ | Num^c^ | A | T | A | G | C | C | G | G | T | C | T | A | *C* | *T* | *A* | *A* | *C* | *C* | *G* | G | G | G | G | C | C | T | T | – | T | C | T | T | T |
| EC 3163 | H7 | H7 | 5 | . | . | . | . | . | . | . | . | . | . | . | . | *.* | *.* | *.* | *.* | *.* | *.* | *.* | . | . | . | . | . | . | . | . | – | . | . | . | . | . |
|  | H7 | R | 1 | . | . | . | . | . | . | . | . | . | . | . | . | *.* | *.* | *.* | *.* | *.* | *.* | *.* | . | . | . | . | . | . | . | . | – | . | . | C | C | A |
|  | H7 | R | 1 | . | . | . | . | . | . | . | . | . | . | . | . | *.* | *.* | *.* | *.* | *.* | *.* | *.* | A | A | C | . | T | T | C | C | A | . | . | C | C | A |
|  | H9 | R | 1 | G | C | C | A | A | A | A | – | – | – | C | G | *T* | *C* | *T* | *G* | *T* | *T* | *T* | A | A | C | . | T | T | C | C | A | . | . | . | . | . |
|  | H9 | H9 | 1 | G | C | C | A | A | A | A | – | – | – | C | G | *T* | *C* | *T* | *G* | *T* | *T* | *T* | A | A | C | . | T | T | C | C | A | . | T | C | C | A |
| EC 3510 | H7 | H7 | 1 | . | . | . | . | . | . | . | . | . | . | . | . | *.* | *.* | *.* | *.* | *.* | *.* | *.* | . | . | . | . | . | . | . | . | – | . | . | . | . | . |
|  | H7 | R | 1 | . | . | . | . | . | . | . | . | . | . | . | . | *.* | *.* | *.* | *.* | *.* | *.* | *T* | A | A | C | . | T | T | . | . | – | . | T | C | C | A |
|  | R | R | 1 | G | . | . | . | . | . | . | – | – | – | C | G | *T* | *C* | *.* | *.* | *.* | *.* | *.* | . | . | . | . | . | . | C | . | – | . | . | . | . | . |
|  | R | R | 1 | G | C | C | A | A | A | A | . | . | . | . | . | *.* | *.* | *T* | *G* | *T* | *T* | *T* | . | . | . | . | . | . | . | . | – | . | . | . | . | A |
|  | H9 | H9 | 1 | G | C | C | A | A | A | A | – | – | – | C | G | *T* | *C* | *.* | *.* | *.* | *T* | *T* | A | A | C | . | T | T | C | C | A | . | T | C | C | A |
|  | H9 | H9 | 1 | G | C | C | A | A | A | A | – | – | – | C | G | *T* | *C* | *T* | *G* | *T* | *T* | *T* | A | A | C | . | T | T | C | C | A | . | T | C | C | A |
| EC 3563 | R | H7 | 1 | G | . | . | . | . | . | . | . | . | . | . | . | *.* | *.* | *.* | *.* | *.* | *.* | *.* | . | . | . | . | . | . | . | . | – | . | . | . | . | . |
|  | H7 | H10 | 1 | . | . | . | . | . | . | . | . | . | . | . | . | *.* | *.* | *.* | *.* | *.* | *.* | *.* | A | A | C | A | T | T | C | C | A | C | T | C | C | A |
|  | H10 | R | 1 | G | C | C | A | A | A | A | – | – | – | C | G | *T* | *C* | *T* | *G* | *T* | *T* | *T* | A | A | C | A | T | T | . | . | – | . | . | . | C | A |
|  | H10 | H10 | 4 | G | C | C | A | A | A | A | – | – | – | C | G | *T* | *C* | *T* | *G* | *T* | *T* | *T* | A | A | C | A | T | T | C | C | A | C | T | C | C | A |
| EC 3655 | H9 | H7 | 1 | G | C | C | A | A | A | A | – | – | – | C | G | *T* | *C* | *T* | *G* | *T* | *.* | *.* | . | . | . | . | . | . | . | . | – | . | . | . | . | . |
|  | H7 | H9 | 1 | . | . | . | . | . | . | . | . | . | . | . | . | *.* | *.* | *.* | *.* | *.* | *.* | *.* | A | A | C | . | T | T | C | C | A | . | T | C | C | A |
|  | H7 | H9 | 1 | . | . | . | . | . | . | . | . | . | . | . | . | *.* | *.* | *T* | *G* | *T* | *T* | *T* | A | A | C | . | T | T | C | C | A | . | T | C | C | A |
|  | H9 | H9 | 1 | N | C | C | A | A | A | A | – | – | – | C | G | *T* | *C* | *T* | *G* | *T* | *T* | *T* | A | A | C | . | T | T | C | C | A | . | T | C | C | A |
| EC 3818 | H7 | H7 | 1 | . | . | . | . | . | . | . | . | . | . | . | . | *.* | *.* | *.* | *.* | *.* | *.* | *.* | . | . | . | . | . | . | . | . | – | . | . | . | . | . |
|  | R | R | 1 | . | . | C | A | A | A | A | – | – | – | C | G | *T* | *C* | *.* | *.* | *.* | *.* | *.* | . | . | . | . | . | . | . | . | – | . | . | C | . | . |
|  | H10 | H7 | 1 | G | C | C | A | A | A | A | – | – | – | C | G | *T* | *C* | *T* | *G* | *T* | *T* | *T* | . | . | . | . | . | . | . | . | – | . | . | . | . | . |
|  | R | H10 | 1 | G | . | N | . | . | . | . | . | . | . | . | . | *.* | *.* | *T* | *G* | *T* | *T* | *T* | A | A | C | A | T | T | C | C | A | C | T | C | C | A |
|  | H10 | H10 | 1 | G | C | C | A | A | A | A | – | – | – | C | G | *T* | *C* | *T* | *G* | *T* | *T* | *T* | A | A | C | A | T | T | C | C | A | C | T | C | C | A |
| POX 102 | H7 | H7 | 4 | . | . | . | . | . | . | . | . | . | . | . | . | *.* | *.* | *.* | *.* | *.* | *.* | *.* | . | . | . | . | . | . | . | . | - | . | . | . | . | . |
|  | H7 | R | 1 | . | . | . | . | . | . | . | . | . | . | . | . | *.* | *.* | *.* | *.* | *.* | *.* | *.* | . | . | . | . | . | . | . | . | - | . | T | C | C | A |
|  | H9 | R | 1 | G | C | C | A | A | A | A | - | - | - | C | G | *T* | *C* | *T* | *G* | *T* | *T* | *T* | A | A | C | . | . | . | . | . | - | . | . | . | . | . |
|  | H9 | H9 | 1 | G | C | C | A | A | A | A | - | - | - | C | G | *T* | *C* | *T* | *G* | *T* | *T* | *T* | A | A | C | . | T | T | C | C | A | . | T | C | C | A |

^a^ Haplotype designation for the IR region of the gene. ‘R’ indicates a recombinant haplotype.

^b^ Haplotype designation for the RAS region of the gene. ‘R’ indicates a recombinant haplotype.

^c^ Number of clones sequenced that had the corresponding haplotype.
