# Supplementary material for: The Plant Pathogen Phytophthora andina Emerged via Hybridization of an Unknown Phytophthora Species and the Irish Potato Famine Pathogen, P. infestans
Source: PLoS One. 2011 Sep 16;6(9):e24543. doi: 10.1371/journal.pone.0024543 (PMC3174952; doi:10.1371/journal.pone.0024543)
Supplement: Table S4 — P. andina haplotypes obtained from cloning just the IR region, including those shown in Table S3. (DOCX) [file pone.0024543.s005.docx]

**Table S4.** *P. andina* haplotypes obtained from cloning just the IR region, including those shown in Table S3.

| Site |  | 21 | 53 | 91 | 95 | 100 | 105 | 112 | 183 | 184 | 185 | 193 | 211 |
| --- | --- | --- | --- | --- | --- | --- | --- | --- | --- | --- | --- | --- | --- |
| Isolate | H^a^ | A | T | A | G | C | C | G | G | T | C | T | A |
| EC 3163 | H7 | . | . | . | . | . | . | . | . | . | . | . | . |
|  | H9 | G | C | C | A | A | A | A | – | – | – | C | G |
| EC 3189 | H7 | . | . | . | . | . | . | . | . | . | . | . | . |
|  | H9 | G | C | C | A | A | A | A | - | - | - | C | G |
| EC 3399 | H7 | . | . | . | . | . | . | . | . | . | . | . | . |
|  | H9 | G | C | C | A | A | A | A | – | – | – | C | G |
| EC 3510 | H7 | . | . | . | . | . | . | . | . | . | . | . | . |
|  | R | G | . | . | . | . | . | . | – | – | – | C | G |
|  | R | G | C | C | A | A | A | A | . | . | . | . | . |
|  | H9 | G | C | C | A | A | A | A | – | – | – | C | G |
| EC 3561 | H7 | . | . | . | . | . | . | . | . | . | . | . | . |
|  | H9 | G | C | C | A | A | A | A | – | – | – | C | G |
| EC 3563 | H7 | . | . | . | . | . | . | . | . | . | . | . | . |
|  | R | G | . | . | . | . | . | . | . | . | . | . | . |
|  | R | G | C | . | . | . | . | . | – | – | – | C | G |
|  | H9 | G | C | C | A | A | A | A | – | – | – | C | G |
| EC 3655 | H7 | . | . | . | . | . | . | . | . | . | . | . | . |
|  | H9 | G | C | C | A | A | A | A | – | – | – | C | G |
| EC 3818 | H7 | . | . | . | . | . | . | . | . | . | . | . | . |
|  | R | . | . | C | A | A | A | A | – | – | – | C | G |
|  | R | G | . | N | . | . | . | . | . | . | . | . | . |
|  | H9 | G | C | C | A | A | A | A | – | – | – | C | G |
| EC 3824 | H7 | . | . | . | . | . | . | . | . | . | . | . | . |
|  | R | G | C | C | A | A | A | A | . | . | . | . | . |
|  | H9 | G | C | C | A | A | A | A | – | – | – | C | G |
| POX 102 | H7 | . | . | . | . | . | . | . | . | . | . | . | . |
|  | H9 | G | C | C | A | A | A | A | – | – | – | C | G |

^a^ Haplotype designation. ‘R’ indicates a recombinant haplotype.
