# Supplementary material for: The Plant Pathogen Phytophthora andina Emerged via Hybridization of an Unknown Phytophthora Species and the Irish Potato Famine Pathogen, P. infestans
Source: PLoS One. 2011 Sep 16;6(9):e24543. doi: 10.1371/journal.pone.0024543 (PMC3174952; doi:10.1371/journal.pone.0024543)
Supplement: Table S5 — P. andina haplotypes obtained from cloning just the RAS region, including those shown in Table S3. (DOCX) [file pone.0024543.s006.docx]

**Table S5.** *P. andina* haplotypes obtained from cloning just the RAS region, including those shown in Table S3.

| Site |  | 512 | 519 | 557 | 569 | 579 | 580 | 657 | 658 | 664 | 692 | 808 | 868 | 869 | 878 |
| --- | --- | --- | --- | --- | --- | --- | --- | --- | --- | --- | --- | --- | --- | --- | --- |
| Isolate | H^a^ | G | G | G | G | C | C | T | T | – | T | C | T | T | T |
| EC 3163 | H7 | . | . | . | . | . | . | . | . | – | . | . | . | . | . |
|  | R | . | . | . | . | . | . | . | . | – | . | . | C | C | A |
|  | R | A | A | C | . | T | T | C | C | A | . | . | C | C | A |
|  | R | A | A | C | . | T | T | C | C | A | . | . | . | . | . |
|  | H9 | A | A | C | . | T | T | C | C | A | . | T | C | C | A |
| EC 3399 | H7 | . | . | . | . | . | . | . | . | – | . | . | . | . | . |
|  | H10 | A | A | C | A | T | T | C | C | A | C | T | C | C | A |
| EC 3510 | H7 | . | . | . | . | . | . | . | . | – | . | . | . | . | . |
|  | R | . | . | . | . | . | . | C | . | – | . | . | . | . | . |
|  | R | . | . | . | . | . | . | . | . | – | . | . | . | . | A |
|  | R | A | A | C | . | T | T | . | . | – | . | T | C | C | A |
|  | H9 | A | A | C | . | T | T | C | C | A | . | T | C | C | A |
| EC 3563 | H7 | . | . | . | . | . | . | . | . | – | . | . | . | . | . |
|  | R | A | A | C | A | T | T | C | C | A | C | . | . | . | . |
|  | R | A | A | C | A | T | T | . | . | – | . | . | . | C | A |
|  | H10 | A | A | C | A | T | T | C | C | A | C | T | C | C | A |
| EC 3655 | H7 | . | . | . | . | . | . | . | . | – | . | . | . | . | . |
|  | H9 | A | A | C | . | T | T | C | C | A | . | T | C | C | A |
| EC 3818 | H7 | . | . | . | . | . | . | . | . | – | . | . | . | . | . |
|  | R | . | . | . | . | . | . | . | . | – | . | . | C | . | . |
|  | H10 | A | A | C | A | T | T | C | C | A | C | T | C | C | A |
| EC 3821 | H7 | . | . | . | . | . | . | . | . | – | . | . | . | . | . |
|  | H10 | A | A | C | A | T | T | C | C | A | C | T | C | C | A |
| POX 102 | H7 | . | . | . | . | . | . | . | . | - | . | . | . | . | . |
|  | R | . | . | . | . | . | . | . | . | - | . | T | C | C | A |
|  | R | A | A | C | . | . | . | . | . | - | . | . | . | . | . |
|  | H9 | A | A | C | . | T | T | C | C | A | . | T | C | C | A |

^a^ Haplotype designation. ‘R’ indicates a recombinant haplotype.
