# Supplementary material for: The Plant Pathogen Phytophthora andina Emerged via Hybridization of an Unknown Phytophthora Species and the Irish Potato Famine Pathogen, P. infestans
Source: PLoS One. 2011 Sep 16;6(9):e24543. doi: 10.1371/journal.pone.0024543 (PMC3174952; doi:10.1371/journal.pone.0024543)
Supplement: Table S6 — P. andina trp1 haplotypes obtained from cloning. (DOCX) [file pone.0024543.s007.docx]

**Table S6.** *P. andina trp1* haplotypes obtained from cloning.

| Site |  |  | 66 | 120 | 169 | 181 | 299 | 329 | 413 | 547 | 552 | 729 |
| --- | --- | --- | --- | --- | --- | --- | --- | --- | --- | --- | --- | --- |
| Isolate | H^a^ | Num^b^ | G | A | T | A | C | G | C | G | A | C |
| EC 3818 | H5 | 1 | . | . | A | C | T | . | . | A | . | . |
|  | R | 1 | A | G | . | . | . | A | T | A | . | . |
|  | R | 1 | A | . | . | . | . | A | T | . | G | T |
|  | H7 | 3 | A | G | . | . | . | A | T | . | G | T |
| POX 102 | H5 | 1 | . | . | A | C | T | . | . | A | . | . |
|  | H7 | 5 | A | G | . | . | . | A | T | . | G | T |

^a^ Haplotype designation. ‘R’ indicates a recombinant haplotype.

^b^ Number of clones sequenced that had the corresponding haplotype.
