# Supplementary material for: The Plant Pathogen Phytophthora andina Emerged via Hybridization of an Unknown Phytophthora Species and the Irish Potato Famine Pathogen, P. infestans
Source: PLoS One. 2011 Sep 16;6(9):e24543. doi: 10.1371/journal.pone.0024543 (PMC3174952; doi:10.1371/journal.pone.0024543)
Supplement: Table S7 — P. andina btub haplotypes obtained from cloning. (DOCX) [file pone.0024543.s008.docx]

**Table S7.** *P. andina btub* haplotypes obtained from cloning.

| Site |  |  | 41 | 70 | 76 | 139 | 167 | 196 | 294 | 333 | 381 | 417 | 543 | 612 | 636 | 669 | 738 | 852 | 961 | 1026 | 1080 | 1152 | 1155 | 1380 | 1571 |
| --- | --- | --- | --- | --- | --- | --- | --- | --- | --- | --- | --- | --- | --- | --- | --- | --- | --- | --- | --- | --- | --- | --- | --- | --- | --- |
| Isolate | H^a^ | Num^b^ | T | T | A | G | G | C | G | G | C | C | T | C | C | C | G | C | C | T | G | A | T | C | G |
| EC 3836 | H10 | 2 | C | . | . | . | . | . | A | . | T | . | C | T | T | T | . | T | T | C | A | C | G | . | T |
|  | R | 1 | . | G | G | A | A | T | . | A | . | T | . | . | . | . | A | . | T | C | A | C | G | . | T |
|  | H11 | 2 | . | G | G | A | A | T | . | A | . | T | . | . | . | . | A | . | . | . | . | . | . | T | . |
| POX 102 | H10 | 2 | C | . | . | . | . | . | A | . | T | . | C | T | T | T | . | T | T | C | A | C | G | . | T |
|  | H11 | 3 | . | G | G | A | A | T | . | A | . | T | . | . | . | . | A | . | . | . | . | . | . | T | . |

^a^ Haplotype designation. ‘R’ indicates a recombinant haplotype.

^b^ Number of clones sequenced that had the corresponding haplotype.
