# Supplementary material for: The Plant Pathogen Phytophthora andina Emerged via Hybridization of an Unknown Phytophthora Species and the Irish Potato Famine Pathogen, P. infestans
Source: PLoS One. 2011 Sep 16;6(9):e24543. doi: 10.1371/journal.pone.0024543 (PMC3174952; doi:10.1371/journal.pone.0024543)
Supplement: Table S8 — P. andina PITG11126 haplotypes obtained from cloning. (DOCX) [file pone.0024543.s009.docx]

**Table S8.** *P. andina* PITG11126 haplotypes obtained from cloning.

| Site |  |  | 61 | 124 | 173 | 258 | 279 | 288 | 325 | 376 | 428 | 435 | 505 | 519 | 523 | 541 | 546 | 612 | 613 | 614 |
| --- | --- | --- | --- | --- | --- | --- | --- | --- | --- | --- | --- | --- | --- | --- | --- | --- | --- | --- | --- | --- |
| Isolate | H^a^ | Num^b^ | T | C | A | A | C | T | * | C | C | A | G | A | C | C | C | A | G | T |
| EC 3510 | H9 | 1 | A | T | G | . | - | C | - | . | T | G | . | T | T | . | . | . | . | . |
|  | H10 | 7 | . | . | . | G | . | . | . | T | . | . | . | . | . | T | G | . | . | . |
| EC 3163 | H8 | 2 | A | T | G | . | - | C | - | . | T | G | A | . | T | . | . | T | C | G |
|  | R | 1 | A | T | G | . | . | . | . | T | . | . | . | . | . | T | G | . | . | . |
|  | H10 | 10 | . | . | . | G | . | . | . | T | . | . | . | . | . | T | G | . | . | . |
| POX 102 | H8 | 1 | A | T | G | . | - | C | - | . | T | G | A | . | T | . | . | T | C | G |
|  | H10 | 3 | . | . | . | G | . | . | . | T | . | . | . | . | . | T | G | . | . | . |

^a^ Haplotype designation. ‘R’ indicates a recombinant haplotype.

^b^ Number of clones sequenced that had the corresponding haplotype.

* 30 bp indel (apparent deletion in H8 and H9)
