# Supplementary material for: The Plant Pathogen Phytophthora andina Emerged via Hybridization of an Unknown Phytophthora Species and the Irish Potato Famine Pathogen, P. infestans
Source: PLoS One. 2011 Sep 16;6(9):e24543. doi: 10.1371/journal.pone.0024543 (PMC3174952; doi:10.1371/journal.pone.0024543)
Supplement: Table S9 — Results of AU and SH tests of tree topologies for ypt1 , btub , and PITG11126. (DOCX) [file pone.0024543.s010.docx]

**Table S9.** Results of AU and SH tests of tree topologies for *ypt1*, *btub*, and PITG11126.

| Topology ^a^ | *ypt1* | *btub* | PITG11126 |
| --- | --- | --- | --- |
| *Pi*,(*Po*,(*Pa*-u,*Pm*)) | **0.431/0.805** | 0.001/0.045 | 0.034/0.104 |
| (*Pi*,*Po*),(*Pa*-u,*Pm*) | **0.660/0.862** | **0.112**/0.093 | 0.034/0.104 |
| *Pi*,(*Pa*-u,(*Pm*,*Po*)) | 0.083/0.060 | 0.025/0.049 | 0.034/0.104 |
| (*Pa*-u,*Pi*),(*Pm*,*Po*) | 0.083/0.060 | 0.025/0.049 | **0.295/0.684** |
| *Pa*-u,(*Pm*,(*Pi*,*Po*)) | 0.083/0.060 | **0.129**/0.079 | 0.034/0.104 |
| *Pm*,(*Po*,(*Pa*-u,*Pi*)) | 0.083/0.062 | 0.009/0.045 | **0.325/0.679** |
| *Po*,(*Pi*,(*Pa*-u,*Pm*)) | **0.602/0.846** | 0.003/0.045 | 0.035/0.104 |
| *Pa*-u,(*Pi*,(*Pm*,*Po*)) | 0.083/0.062 | 0.052/0.041 | 0.035/0.104 |
| *Pi*,(*Pm*,(*Pa*-u,*Pi*)) | 0.083/0.063 | 0.042/0.050 | **0.204/0.133** |
| *Pm*,(*Pi*,(*Pa*-u,*Pi*)) | 0.067/0.063 | 0.009/0.045 | **0.212/0.127** |
| (*Pi*,*Pm*),(*Pa*-u,*Pi*) | 0.067/0.063 | 0.042/0.050 | **0.204/0.133** |
| *Po*,(*Pm*,(*Pa*-u,*Pi*)) | 0.077/0.070 | 0.042/0.050 | **0.895/0.952** |
| *Pa*-u,(*Po*,(*Pi*,*Pm*)) | 0.083/0.062 | 0.050/0.040 | 0.034/0.104 |
| *Po*,(*Pa*-u,(*Pi*,*Pm*)) | 0.075/0.070 | 0.041/0.050 | 0.035/0.104 |
| *Pa*-u,(*Pm*,(*Pi*,*Po*)) | 0.083/0.062 | **0.107**/0.096 | 0.033/0.099 |
| (*Pi*,*Po*),(*Pm*,(*Pa*-u,*Pm*))^b^ | - | **0.873/0.920** | - |
| *Po*,(*Pi*,(*Pm*,(*Pa*-u,*Pm*)))^b^ | - | **0.250/0.610** | - |
| *Pi*,(*Po*,(*Pm*,(*Pa*-u,*Pm*)))^b^ | - | **0.353/0.615** | - |

^a^ All trees were rooted with *P. phaseoli*. Species names are abbreviated as follows: *Pi* - *P. infestans; Pm* - *P. mirabilis*; *Po* - *P. ipomoeae*. *Pa*-u indicates the non-*P. infestans* (unknown) parent of the hybrid *P. andina*.

^b^ In these trees *P. mirabilis* is not monophyletic, with topology as shown in Fig. 2C.
